# Comparative physicochemical and structural characterisation studies establish high biosimilarity between BGL-ASP and reference insulin aspart

**DOI:** 10.1038/s41598-024-54819-x

**Published:** 2024-02-20

**Authors:** Nikhil S. Ghade, Damodar K. Thappa, Jeseena Lona, Archana R. Krishnan, Sanjay M. Sonar

**Affiliations:** BioGenomics Limited, Thane, Maharashtra 400610 India

**Keywords:** Biotechnology, Biological techniques, Peptide hormones

## Abstract

Biosimilar insulin analogues are increasing market access for diabetic patients globally. Scientific establishment of biosimilarity is cornerstone of this key change in the medical landscape. BGL-ASP is a biosimilar insulin aspart developed by BioGenomics Limited, India. BioGenomics has considered a stepwise approach in generating the totality of evidence required to establish similarity with reference product. Insulin aspart is a recombinant rapid-acting human insulin analogue utilised in the treatment of type-1 and type-2 diabetes mellitus. The single amino acid substitution at position B28 where proline is replaced with aspartic acid results in a decreased propensity to form hexamers, thus increasing the absorption rate on subcutaneous administration compared to native insulin. In order to establish the safety and efficacy of BGL-ASP, the critical quality attributes (CQAs) of BGL-ASP are identified based on the impact created on biological activity, pharmacokinetic/pharmacodynamic (PK/PD), immunogenicity and safety. The CQAs of insulin aspart are related to product structure, purity and functionality and are characterised using a series of state-of-the-art orthogonal analytical tools. The primary protein sequence, the secondary, tertiary and quaternary structure are found to be highly similar for BGL-ASP and reference product. The product related impurities of insulin aspart and the assay content are determined using high performance liquid chromatography (HPLC) based analysis and is similar for BGL-ASP and reference insulin aspart sourced from United States of America (US), Europe Union (EU) and India. The safety, efficacy and immunogenicity of BGL-ASP is also found to be comparable with reference product and is confirmed through the clinical trials conducted as recommended by International Council for Harmonisation of Technical Requirements of Pharmaceuticals for Human Use (ICH) and European Medicines Agency (EMA) guidelines. The data encompassed in this study demonstrates that reference insulin aspart and BGL-ASP are highly similar in terms of structural, physicochemical, and biological properties, thus confirming its safety and efficacy for usage as potential alternative economical medicinal treatment for diabetes mellitus.

## Introduction

The discovery of insulin has completed 100 years, however, the accessibility and affordability of the molecule to patients is still a major concern, especially individuals in the low-income group worldwide. Based on the reported data it is observed that in individuals with type 2 diabetes, the access is restricted to 50% patients globally and 15% patients in the sub-Saharan Africa^1^. As the cases of type 2 diabetes are increasing annually, international guidelines prefer insulin analogues to human insulin because of lesser weight gain, better HbA1C control and effectivity^2^. However, the high cost of these products become a deterrent in usability, resulting in either terminating the treatment or resorting to lower usage than the prescribed dose.

The manufacturing of insulin and analogues are limited to two–three large companies creating monopoly with respect to the product pricing. In order to reduce the product cost and enhance the accessibility and affordability of insulins, it is imperative that newer manufacturers introduce safe and efficacious product manufactured in a GMP-compliant facility to increase the competition. Thus, to make affordable treatment on controlling blood glucose levels accessible to diabetes patients, BioGenomics has developed BGL-ASP, a biosimilar developed in reference to the marketed insulin aspart 100 units/ml (NovoRapid by Novo Nordisk A/S, Denmark).

Insulin aspart is a recombinant rapid-acting human insulin analogue utilized in the treatment of type-1 and type-2 diabetes mellitus. It is an analogue of human insulin made by replacing the proline residue at position B28 of the B-chain with aspartic acid^3,4^. The switch in amino acids results in a decreased tendency of the insulin aspart to form hexamers, thus enhancing the rate of absorption from the subcutaneous injection sites. The properties of insulin aspart are suitable for “basal-bolus” regimen representing a more physiological plasma insulin profile, in which basal insulin concentrations are provided by long-acting insulin preparations and bolus or high levels of insulin are provided by rapid-acting insulin analogues^5,6^.

The production of insulin aspart by innovator is undertaken in *Saccharomyces cerevisiae* while BGL-ASP is manufactured in a non-pathogenic strain of *Escherichia coli*^4^. As the manufacturing processes of insulin aspart are different for BGL-ASP and reference product there are possibilities of differences arising in quality attributes and these comparisons are initiated in the early phase of development prior to clinical trials. The objective of the comparative data analysis is to demonstrate that the critical quality attributes of BGL-ASP and reference product are similar even though the cultivation systems and manufacturing processes are different^7^.

**Figure 1:**
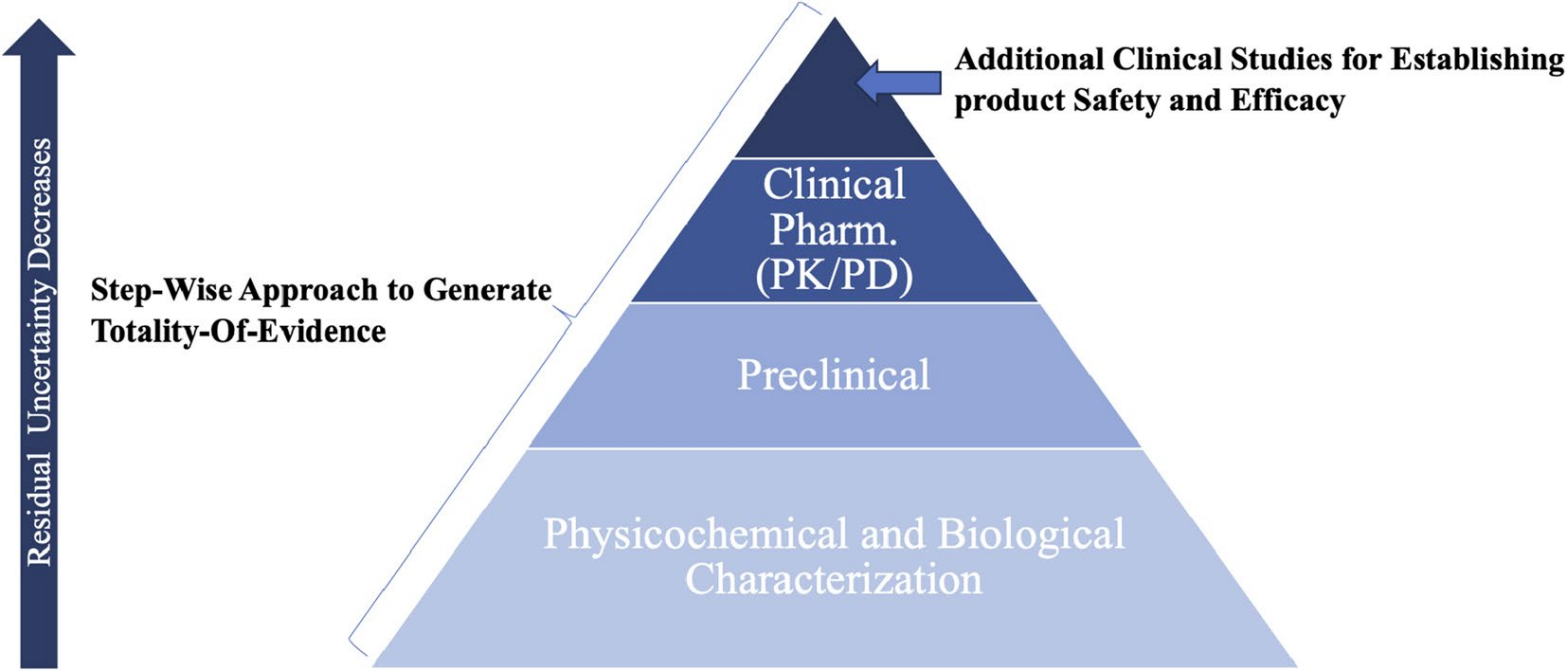
Development pathway considered for establishing biosimilarity of BGL-ASP. The development pathway demonstrates additional emphasis on the physicochemical and biological characterization data to establish high level of similarity between BGL-ASP and reference product. The phase I and phase III clinical trial data is important to reduce the residual uncertainty^9,10^.

BioGenomics has considered a stepwise approach in generating the totality of evidence required to establish similarity with reference product as shown in Fig. 1^8^. In the first step the focus was on establishing comprehensive structural, physicochemical and biological similarity between reference product and BGL-ASP using state-of-the-art orthogonal analytical tools. These studies were highly sensitive, and the aim was to detect minor difference in quality attributes of clinical relevance between reference product and BGL-ASP. The characterization studies demonstrated a high similarity in the critical quality attributes of BGL-ASP and reference product.

The second step involved comparative, pre-clinical studies comprising of: four single dose, two repeat dose and one skin sensitization studies. The repeat dose studies were conducted in comparison with reference product and no abnormal clinical signs or adverse effects were observed post studies. In the third step bioequivalence of pharmacokinetic (PK) and pharmacodynamic (PD) parameters were demonstrated for BGL-ASP and reference product through phase I clinical studies. The EMA-compliant phase III clinical studies demonstrated that the immunogenicity profiles and drop in HbA1c values were identical for Type 2 (T2DM) diabetes mellitus patients treated with BGL-ASP and reference product, thus confirming the product safety, efficacy and immunogenicity^11^.

In this report, the data related to structural and physicochemical characterization of insulin aspart is presented. Functional characterization studies such as cell-based glucose uptake, Insulin Receptor-B (IR-B) phosphorylation, lipogenesis, in vivo rabbit bioidentity tests and mitogenicity as an off-target assay for BGL-ASP have been completed in comparison with the reference product and the results were found to be highly similar, however, these studies are beyond the scope of this paper and are published separately^12^.

### Risk ranking of quality attributes

Based on the analysis of multiple batches of reference insulin aspart, the quality target product profile (QTPP) was determined. The QTPP assisted in understanding the attributes related to product quality, safety and efficacy and formed the basis for identifying critical quality attributes (CQAs). The CQAs assisted in developing a control strategy for the manufacturing process essential in controlling the product quality throughout its lifecycle. The risk ranking of quality attributes for insulin aspart was assessed based on the impact created on safety and efficacy. The criticality score for a quality attribute was calculated as a function of impact and uncertainty^13,14^. The impact score (such as very high, high, moderate or low) was calculated based on the impact created on biological activity, pharmacokinetics (PK), pharmacodynamics (PD), immunogenicity and safety^15^. The uncertainty score was based on the either the literature available for an impurity or the presence of the variant in material used for clinical studies. The Table 1 below demonstrates the varied quality attributes and the classification of criticality. The categorization of the attributes was performed as per the principles and concept mentioned in International Council for Harmonisation (ICH) Q8 and ICH Q9.

**Table 1:** Risk ranking of quality attributes and testing methods.

Test Classification	Quality Attributes	Methods	Criticality
Primary Structure	Amino Acid Sequence	Reduced peptide mass fingerprinting using Trypsin (LC–MS/MS)	Very High
	Molecular Weight	Intact protein molecular mass analysis (LC–MS)	
	Disulphide Linkage	Reduced peptide mass fingerprinting using V8 enzyme (LC–MS/MS)	
		Non-reduced peptide mass fingerprinting (LC–MS/MS)	
Higher Order Structure	Secondary Structure	Far UV-CD Spectroscopy	High
		FTIR Spectroscopy	
	Tertiary Structure	NMR Spectroscopy	
		Near UV-CD Spectroscopy	
		Fluorescence Spectroscopy	
	Quaternary Structure	Differential Scanning Calorimetry (DSC)	
Strength	Concentration	RP-HPLC–UV	High
Product Related Impurities	Higher Molecular Weight Protein (HMWP)	SE-HPLC–UV	High
	Impurities such as deamidated, oxidized and isomerized forms	RP-HPLC–UV	High
Formulation Components	Phenol Content	RP-HPLC based quantitation	Moderate
	M-cresol Content	RP-HPLC based quantitation	Moderate
	Zinc	Atomic Absorption Spectroscopy	Moderate
	pH	Potentiometry	Moderate
Additional Characterization Tests not related to Biosimilarity	Host Cell Protein (HCP)	ELISA	Considered critical to product safety and immunogenicity but not related to biosimilarity
	Host Cell DNA (HCDNA)		
	Single Chain Precursor (SCP)		
	Residual Enzyme Content		
	Residual Solvent Content	GC–MS based analysis	

LC–MS/MS, liquid chromatography tandem mass spectrometry; UV-CD, ultraviolet-circular dichroism; FTIR, fourier transform infrared spectroscopy; NMR, nuclear magnetic resonance;RP-HPLC–UV, reverse phase-high performance liquid chromatography-ultraviolet; SE-HPLC–UV, size exclusion-high performance liquid chromatography-UV; ELISA, enzyme-linked immuno sorbent assay;GC–MS, gas chromatography-mass spectrometry.

The section 351(i) of the Public Health Service (PHS) Act of United States Food and Drug Administration (USFDA) defines biosimilarity to mean “that the biological product is highly similar to the reference product notwithstanding minor differences in clinically inactive components” and that “there are no clinically meaningful differences between the biological product and the reference product in terms of the safety, purity, and potency of the product”^16^. The similarity assessment studies were performed in congruence with the regulatory guidelines using the developed scientific understanding of the molecule.

As mentioned in Table 1, the amino acid sequence and the native disulphide linkages are the basic elements governing the function and safety of insulin aspart, hence its criticality was considered as very high. The criticality was assigned as high to structural attributes related to pharmacological activity, protein concentration determined by RP-HPLC in relation to dosage accuracy and product related impurities. The deamidated, oxidized and isomerized forms are considered as triggers for Higher Molecular Weight Protein (HMWP) formation^17^. The HMWP are inactive forms of insulin and hence the criticality was considered as high. Insulin Aspart binds strongly to the stationary phase of cation exchange chromatography and capillary electrophoresis and protein elution and separation does not occur under recommended chromatography or electrophoresis conditions. Hence the product related impurities are identified using RP-HPLC based analysis. The preservatives, pH, and zinc content are important elements of insulin aspart formulation related to desired in-solution structure of insulin aspart and impacts product efficacy. However, these formulation excipients are found to be effective over a wide range and hence their criticality was considered as moderate^18–21^. In the formulation of reference product and BGL-ASP, glycerol and sodium chloride are added as tonicity agents, hydrochloric acid/ sodium hydroxide are used for pH adjustment and disodium phosphate dihydrate is used as buffering agent. The excipient grade quality of these chemicals used in formulation ensures no major impact on the product efficacy and safety and hence these chemicals are not documented in Table 1. The HCP, HCDNA, SCP, residual enzyme, residual solvent and residual moisture content are important quality attributes related to product safety, however, these impurities are present in low quantities in the product and hence the criticality was considered as low. Additionally, these impurities are process-specific and hence not considered as part of biosimilarity^22^.

### Product related impurities of insulin aspart

**Figure 2:**
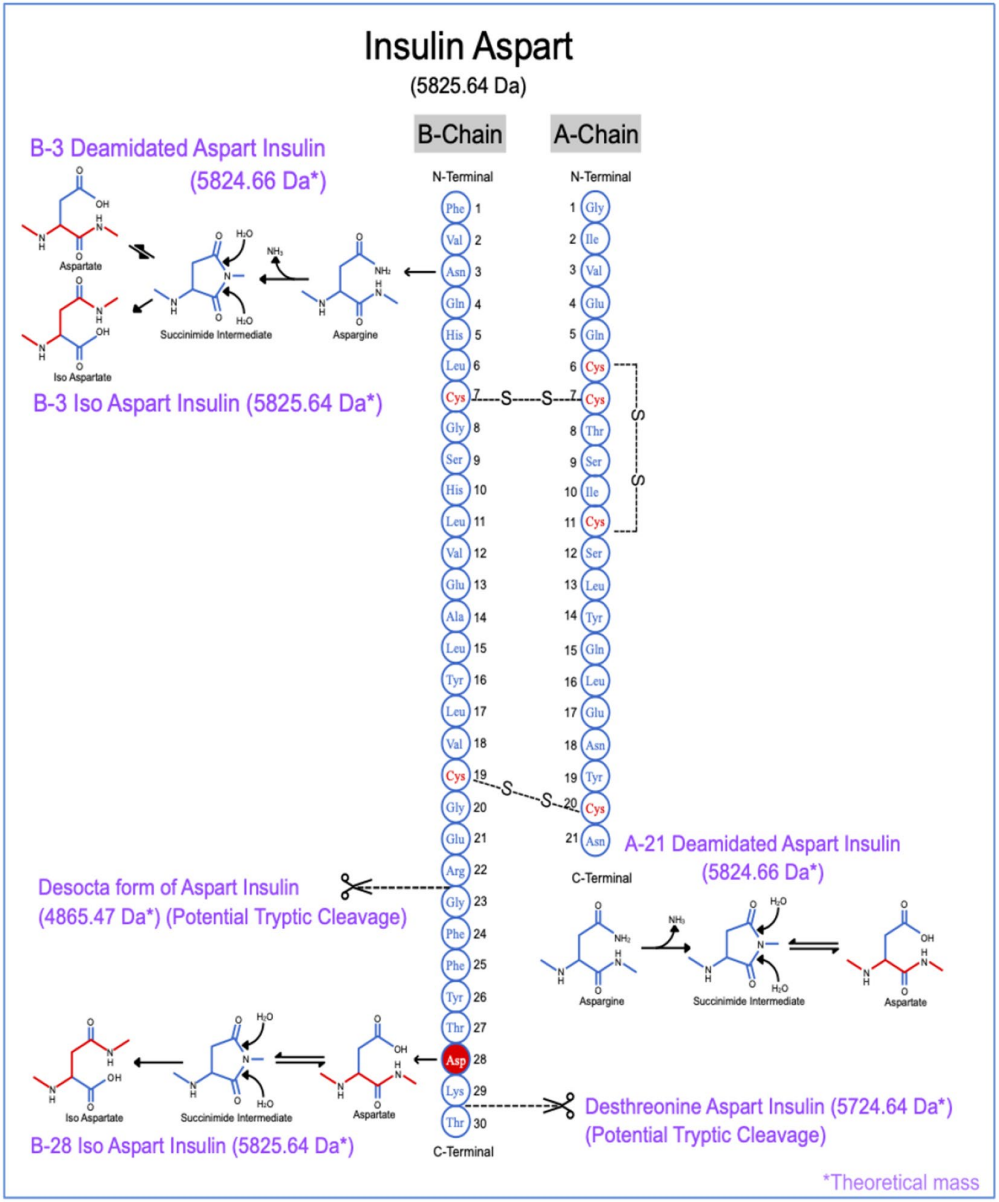
Product related impurities and potential tryptic cut sites for insulin aspart. The figure demonstrates the location of the native disulphide linkages in insulin aspart and position of amino acids prone to degradation. The protease Trypsin specifically cleaves at the carboxyl side of the amino acids lysine and arginine and the potential cut sites in insulin aspart are shown in the figure.

The asparagine (Asn) at position A21 (A21Asn) and B3 (B3Asn), and aspartic acid (Asp) at B28 (B28Asp) position are susceptible to degradation resulting in the formation of product related impurities as shown in Fig. 2. The Asn at A21 is susceptible to deamidation under acidic conditions and under neutral conditions insulin deamidates at Asn at B3 position, resulting in the formation of AspB3 and isoAspB3 as the main products. The neutral formulation condition additionally initiates the isomerization of Asp at B28 position^23^. The asparagine (Asn) deamidation and aspartate (Asp) isomerization reactions are non-enzymatic, intra-molecular rearrangement reactions occurring in peptides and proteins, and these non-native amino acid residues are a source of major stability concern in the formulation of these biomolecules.

Additionally, the other product related impurity observed with insulin aspart is the presence of higher molecular weight protein (HMWP)^24^. The HMWP formation typically occurs due to intermolecular disulphide bonds.

## Material and methods

### Manufacturing of BGL-ASP

The manufacturing of insulin aspart incorporates the utilization of the bacterial cultivation system (BCS). The BCS provides distinct advantages such as absence of post-translational modifications and fast growth kinetics. The disadvantage is the formation of the inactive inclusion bodies with non-native conformation. The developmental work involved rigorous optimisation of refolding/renaturation conditions that generates native disulphide linkage and conformation^25–32^.

Insulin aspart is expressed in its precursor form as inclusion bodies in a non-pathogenic laboratory strain of *E.coli* as the production organism. The laboratory strain is classified as non-hazardous by the 2012 Occupational Safety and Health Administration (OSHA) Hazard Communication Standard (29 CFR 1910.1200) and regulation (EC) 1272/2008 [CLP]. The seed culture is prepared from a characterized and qualified working cell bank. The seed flasks are transferred to the inoculums flasks on achieving the desired growth criteria. The inoculum is further added to the seed fermenter, which acts as feed source for the production fermenter. The production fermenter is operated as per the predefined conditions and the insulin aspart precursor (IAP) is overexpressed in the presence of suitable inducers at optimal concentrations and temperature. The overexpression of insulin aspart precursor results in the formation of inclusion bodies. The cell harvest is isolated using continuous centrifugation and subjected to cell-lysis using high pressure homogenization systems. The inclusion bodies are dense particles and are isolated from the cell debris through a series of centrifugation cycles. The isolated inclusion bodies of IAP are subjected to the process of renaturation resulting in the native conformation with expected disulphide linkages. The IAP is converted to insulin aspart in the presence of recombinant enzymes such as Trypsin and Carboxypeptidase B^31,32^. The purification process involves low pressure and high pressure-based chromatography for separation of process and product related impurities and generation of a purified form of insulin aspart qualifying as per the predefined acceptance criteria^33^. The insulin aspart drug substance (DS) is manufactured in the freeze-dried form and consists of salt-free anhydrous insulin aspart. The DS is formulated in the presence of pharmacopeial grade excipients such as phenol, m-cresol, glycerol, zinc chloride, disodium phosphate dihydrate, sodium chloride, hydrochloric acid (for pH adjustment), sodium hydroxide (for pH adjustment) and water for injections, at a concentration of 3.5 mg/ml equivalent to 100 units/ml. The formulated DS is referred to as BGL-ASP in this study.

The placebo was prepared by weighing 75 mg of phenol (Merck, Cat No: 1.00201) and 86 mg of m-cresol (Finar Chemicals) in a 50 ml beaker. 15 ml of purified water was added in the beaker and mixed thoroughly. 800 mg of glycerol (Finar Chemicals) was added in another 50 ml beaker and mixed with 15 ml of purified water. The phenol and m-cresol solution was mixed with the glycerol solution and 204 µl of zinc chloride (Merck, Cat No: 1.28221) from a stock solution of 10 mg/ml is added. Further to this solution, 29 mg of sodium chloride (Merck, Cat No: 1.16224) and 62.65 mg of di-sodium hydrogen phosphate (Merck, Cat No: 1.06585) was added and dissolved completely. The pH of solution was adjusted to 7.4 using 10% sodium hydroxide (Merck, Cat No: 1.37020). The volume was adjusted to 50 ml with purified water and the solution was filtered with a 0.22 µm filter.

For spectroscopic techniques such as CD, Fluorescence and NMR, the drug substance was extracted from the drug product using isoelectric precipitation methodology. The pH of the drug product was adjusted to 5.0 ± 0.1 using glacial acetic acid (Cat No: 85801(0129168), Sisco Research Laboratories) and the precipitate was allowed to form for 30 min. The precipitate was isolated by centrifugation at 10,000 g for 30 min using the Sorvall Legend XFR centrifuge (Thermo Fisher Scientific). The temperature was maintained at 2–8 °C during the cetrifugation cycle. The isolated precipitate was washed twice with cold water for injection. The washed precipitate was recontistuted in 10 mN hydrochloric acid at appropriate concentration for spectroscopic analysis. The protein concentration of insulin aspart was determined using extinction coefficient of 1.09(mg/ml)^−1^ cm^−1^.

### Physicochemical and structural characterization studies

The physicochemical characterisation of insulin aspart involves tests related to purity, assay content, HMWP content, preservatives and zinc content. Comprehensive structural characterisation of insulin aspart was performed using techniques such as NMR, FTIR, CD, DSC and intrinsic fluorescence spectroscopy. The peptide mass fingerprinting under reduced and non-reduced conditions and mass analysis was performed using LC–MS technique. The process related impurities such as HCP, HCDNA, residual enzyme, SCP were also analyzed and the data for the same was recorded. The N-terminal and C-terminal sequencing using LC–MS/MS technique, sodium dodecyl-sulfate polyacrylamide gel electrophoresis (SDS PAGE)^34^ and western blot analysis^35^ was also performed (data not shown).

#### Reference product details

The reference product formulation contains insulin aspart 100 Units/ml, glycerin 16 mg/ml, phenol 1.50 mg/ml, metacresol 1.72 mg/ml, zinc 19.6 mcg/ml, disodium hydrogen phosphate dihydrate 1.25 mg/ml, sodium chloride 0.58 mg/ml and water for injection. The pH of reference product is in the range of 7.2–7.6. Hydrochloric acid 10% and/or sodium hydroxide 10% may be added to adjust pH^36^. The reference product batches were sourced from India, Europe and United States of America (USA). The European Pharmacopeia Commission of Reference Substance (EPCRS) for insulin aspart manufactured by European Directorate for the Quality of Medicines and HealthCare (EDQM) was procured from Sigma-Aldrich (Cat No. Y0000349).

#### RP-HPLC based analysis of insulin aspart and quantitation of preservative content

The related impurities of insulin aspart were monitored using RP-HPLC based analysis. The column used for HPLC analysis was an octadecylsilyl silica gel column procured from Kromasil (250 mm × 4 mm; 5 µm) (Cat No: E118882). The mobile phase A was prepared by dissolving 142.0 g of anhydrous sulfate, reagentplus grade (Cat. No. 59977, Sisco Research Laboratories) in water and further addition of 13.5 ml of phosphoric acid (Cat No. 1.93403.0521/79606, Sigma-Aldrich). The volume was adjusted to 5000 ml with water and pH was adjusted to 3.6 with sodium hydroxide (Cat. No. 1.93502.0521, Merck). Nine volumes of this solution was merged with one volume of acetonitrile (Cat No. 61803025001730, Merck). The mobile phase B was prepared by mixing equal volume of acetonitrile and water. The separation was performed at 40 °C, at a flow rate of 1 ml/min, with a total run time of 60 min. The gradient details are mentioned in Table 2.

**Table 2:** Gradient details for RP-HPLC based separation of related impurities.

Time (min)	Mobile phase A (per cent V/V)	Mobile phase B (per cent V/V)
0–35	58	42
35–40	58→20	42→80
40–45	20	80
45–46	20→58	80→42
46–60	58	42

The monitoring wavelength was set at 214 nm. The injection volume was 10 μl. The RP-HPLC method can distinctly separate related impurities of insulin aspart such as B28isoAsp, B3Asp, A21Asp and B3isoAsp. The method was also utilized to calculate assay content against insulin aspart chemical reference standard from European Pharmacopeia (EP) (Order Code: Y0000349)^37^.

The RP-HPLC methodology was also utilized to quantitate phenol and m-cresol content in BGL-ASP and reference product formulations. 100 µl of BGL-ASP (100 U/ml) was taken in a microfuge tube and 900 µl of purified water was added. The solution was acidified with 4 µl of 6 M hydrochloric acid and 10 µl of this solution was used as injection volume. A similar process was repeated for reference product (100 U/ml) formulation. The quantitation of the phenol content was performed using United States Pharmacopeia (USP) reference standard for phenol (Cat No: 1524806) and metacresol USP reference standard (Cat No: 1395204).

#### Peptide mass fingerprinting—LC–MS based analysis of intact, reduced and V8 enzyme digested peptides of insulin aspart

In order to demonstrate that the disulphide linkage in BGL-ASP batches were highly similar to reference insulin aspart, a comparative peptide mapping analysis was performed wherein insulin aspart was digested with V8/Endoproteinase GluC (Cat no: P2922, Sigma-Aldrich) enzyme and the mixture generated was analyzed under reducing and non-reducing conditions using Liquid Chromatography-Electron Spray Ionization-Mass Spectrometry/Mass Spectrometry (LC–ESI–MS/MS). The reduction reaction was set up in the presence of Dithiothreitol (DTT) (Cat No: 10197777001, Sigma-Aldrich). The comparison between the theoretical and generated molecular weights for the peptides, assisted in demonstrating the presence of the native disulphide linkages. The untreated insulin aspart mass was also determined and compared with the innovator product. The column with the dimensions C18, 150 × 2.1 mm, 3 μm, (Cat No: TA12S05-15Q1PTH, YMC) was used for LC–MS based analysis. The detection wavelength was set at 215 nm, flow rate of 0.2 ml/min. The AB SCIEX Triple TOF 4600 was set to mass range of m/z 200–2000 Da, source temperature of 450 °C and collision energy of 35 V.

#### SE-HPLC based analysis of higher molecular weight proteins (HMWP) in insulin aspart

The HMWP content in insulin aspart was determined using SE-HPLC based analysis. The column used for SE-HPLC analysis was a hydrophilic silica gel column procured from Waters (7.8 × 300 mm; 5 µm) (Cat No: WAT201549). The mobile phase was prepared by mixing glacial acetic acid (Cat No: 85801(0129168), Sisco Research Laboratories), acetonitrile (Cat No: 61803025001730/A2094, Merck), and 1.0 g/l solution of arginine, reagentplus grade (Cat No: 014879, Sisco Research Laboratories) in a ratio of 15:20:65. Isocratic elution was performed at a flow rate of 0.5 ml/min at 25 ºC, with a total run time of 35 min. The monitoring wavelength was set at 276 nm. The sample and reference solution were acidified and injected at a concentration of 100 units/ml^37^.

#### Stability studies

The stability studies of BGL-ASP are performed as per International Conference on Harmonization (ICH) guidelines documented in ICH Q5C. The stability studies for BGL-ASP are performed for attributes such as assay, HMWP and related impurities content that are susceptible to change during storage and are likely to influence quality safety and/or efficacy. The stability studies for BGL-ASP such as accelerated stability, forced degradation studies and in-use stability studies are performed in comparison with the reference product. The Table 3 demonstrates the conditions for the varied stability studies.

**Table 3:** Conditions for stability studies.

Stability studies	Conditions	Duration	Intervals	Techniques Utilized for analysis
Accelerated stability studies	30 °C ± 2 °C, 70% RH ± 5% RH	6 month	0, 1, 2, 3 and 6 month	SE-HPLC based analysis of HMWP content RP-HPLC based analysis of assay and related impurities content
Forced degradation studies	50 °C ± 2 °C, 75% RH ± 5% RH	14 days	0, 5, 7 and 14 days	
In-use stability studies	30 °C ± 2 °C, 70% RH ± 5% RH	28 days	1, 7, 14, 21 and 28 days	

#### Zinc content estimation

The zinc content estimation was performed using atomic absorption spectrophotometer (Lab India, India, AA8000). Zinc chloride (Merck, Cat No: 1.28221) reference standard solution was prepared at a concentration of 5 mg/ml. The stock solution was utilized to prepare a standard curve in the range of 0.2–1.0 µg of zinc per ml using 10 mN hydrochloric acid as diluent. The test solution was prepared by diluting a volume containing 100 units of insulin aspart to 25 ml with 10 mN hydrochloric acid. The instrument was set at 213.9 nm in source of zinc hollow- cathode lamp and atomization device air-acetylene flame. The absorbance of the standard and sample solution were measured and recorded. The zinc content in aspart formulation was determined using the standard curve.

#### Nuclear magnetic resonance (NMR) spectroscopy

The NMR spectroscopy was performed using Bruker 800 MHz NMR spectrometer (Bruker Biospin, Switzerland, Avance AV 800) at 25 °C. The spectrometer was equipped with gradient 5.0 mm probe. The data acquisitions and processing were carried out using Topspin 4.1.4. The NMR spectroscopy was performed using drug substance of insulin aspart extracted from formulation of BGL-ASP and reference product. The drug substance of BGL-ASP and reference product were initially reconstituted in 10 mN HCl at a concentration of 10 mg/ml. The stock solution of drug substance was diluted with acetonitrile-d3 to achieve a final concentration of 7 mg/ml and utilized for NMR based analysis. The reconstituted sample was utilized to obtained proton 1D, COSY, TOCSY, NOESY and HSQC spectra. The comparison of the spectra was performed to establish similarity between BGL-ASP and reference product. The acquisition parameters for NMR experiments are listed in the Table 4.

**Table 4:** NMR acquisition parameters.

Experimental name	Encoded Nucleus		Complex points		Spectral width (Hz)		Carrier offset (ppm)		Number of scans, notes
	F1	F2	F1	F2	SW1	SW2	1H	13C	
Proton 1D	^1^H	–	16,384	–	9615.385	–	4.392	–	64
2D ^1^H-^1^H COSY	^1^H	^1^H	2048	512	9615.385	9615.385	4.385	–	16
2D ^1^H-^1^H TOCSY	^1^H	^1^H	4096	512	9615.385	9615.385	4.385	–	32, mixing time of 80 ms
2D ^1^H-^1^H NOESY	^1^H	^1^H	4096	512	9615.385	9615.385	4.385	–	48, mixing time of 250 ms
2D ^1^H-^13^C HSQC	^1^H	^13^C	2048	256	9615.385	33,195.160	4.382	75.009	64

#### Fourier-transformed infrared (FTIR) spectroscopy

The FTIR analysis was performed using FT-IR ALPHA II spectrometer from Bruker using attenuated total reflection (ATR) mode, equipped with software OPUS. The BGL-ASP and reference product were subjected to FTIR analysis at a concentration of 3.5 mg/ml, with the IR spectra recorded between 4000 and 600 cm^−1^, with resolution set at 4 cm^−1^. Each spectrum was averaged over 32 scans and five such spectra were recorded for each sample.

#### UV circular dichroism spectroscopy

The UV circular dichroism (UV-CD) spectroscopy based analysis was used to study tertiary and secondary structural elements of insulin aspart. The UV-CD experiments were performed using Jasco J-815 spectrometer. The circular dichroism (CD) spectra in the near-UV region (250–350 nm) and the far-UV region (190–250 nm) were recorded at a concentration of 0.2 mg/ml. The far-UV spectra were recorded with path length of 0.1 cm and bandwidth of 0.1 nm in a quartz cell, at 25 °C with a scanning speed of 200 nm/min. For recording the spectra, 6 accumulations were performed for each sample. The near-UV spectra were recorded at a path length of 1 cm. The baseline subtraction was performed by subtracting the buffer signal from the spectrum of the sample^38^.

#### Intrinsic fluorescence spectroscopy

Intrinsic fluorescence spectroscopy-based analysis was performed using Synergy H1 hybrid Multi-mode reader (Biotek instruments). Intrinsic fluorescence analysis was performed at a protein concentration of 1 mg/ml by excitation at 278 nm and emission was scanned from 300 to 400 nm. The fluorescence emission spectrum were recorded by subtraction of the blank buffer emission spectra values^38^.

#### Differential scanning calorimetry (DSC)

Differential scanning calorimetry based analysis was performed using NanoDSC from TA Instruments, New Castle, DE. The instrument was equipped with DSCrun and NanoAnalyzer software. The thermal stability measurements were performed in the range of 20–100 °C at ramp rate of 1.5 °C/min. The BGL-ASP and reference product were subjected to analysis without any dilution. The placebo was used as control. The generated thermograms were compared by Pearson’s correlation testing.

#### Bacterial endotoxin test (BET)

The bacterial endotoxin content was determined using the gel clot method. 10 ng vial of endotoxin was reconstituted with 5.0 ml Limulus Amebocyte Lysate (LAL) reagent water (Charles River). The endotoxin was diluted with LAL reagent water to a concentration of 1 EU/ml. The Limulus Amebocyte Lysate was prepared by reconstituting the lyophilized lysate in LAL reagent water. Depyrogenated auto pipettes with depyrogenated tips were used for all dilutions and sample transfers. The pH of the sample was adjusted to approximately 7.4 using 0.1 N HCl or 0.1 N NaOH. The sample and lysate were mixed by slight shaking and the tubes were immediately incubated in waterbath at 37 ± 1 °C for 60 ± 2 min. After incubation, the formation of gel clot was checked by inverting the tubes. The analysis of each sample was performed in triplicates. The system suitability included the formation of gel clot in the positive sample and the absence of clot in the negative sample^39,40^.

#### Loss on drying (LOD)

The residual moisture content in insulin aspart DS was determined using the loss on drying (LOD) method. The test is based on the thermogravimetric principle, wherein the substance is heated till it is completely dried and no more weight is lost. The insulin aspart DS was placed in a dry a clean glass-stoppered, shallow weighing bottle. The oven temperature was set at 105 ± 2 °C and the substance was incubated in it for 24 h. The bottle was weighed after drying and based on the weight difference the percentage LOD was identified.

#### Host cell protein (HCP)

The *E. coli* HCP ELISA Detection Kit detects HCP in test samples by double-antibody sandwich method. The standard and test samples were added to reaction wells on microplate pre-coated with antibody for incubation. The present HCP quantitatively binds to the antibodies in the microplate, while unbound substances were removed after plate washing. Anti-HCP-Biotin and Streptavidin HRP were added successively to form an antibody—antigen—biotinylated antibody—enzyme-labeled avidin complex. 100 µl/well of the substrate was added and incubated at 37 °C. The plate was read at 630 nm using the Spectramax i3x plate reader (Molecular Devices, USA) from 30 to 120 min. The data generated by the software SoftMax Pro (Molecular Devices, USA) was further used for analysis and calculations.

#### Host cell DNA (HCDNA)

The quantitative detection of residual Host Cell DNA (HCDNA) was performed using Real-Time Polymerase Chain Reaction (RT-PCR) using CFX Connect (BIO-RAD, USA). The initial heat denaturation was performed at 95 °C for 7 min, followed by 30 cycles each of 95 °C for 10 s and 65 °C for 30 s. All reactions were run in triplicate^41^.

#### Residual enzyme analysis

The residual enzymes in insulin aspart were detected using a highly sensitive enzyme linked immunosorbent assay (ELISA). The ELISA was initiated by overnight coating of high binding 96 wells with 100 µl (2.0 µg/ml) of capture antibody. The wells coated with capture antibody was washed with phosphate buffered saline with tween (PBST) after overnight incubation. The non-specific sites of the coated wells were blocked for 60 min at 22–24 °C with 100 µl of blocking buffer (3% BSA in PBST). The wells were washed with PBST and then 100 µl of standard or sample solution was added to respective wells of assay plate and incubated at 37 °C for 45 min. The assay plate was washed with PBST and incubated with 100 µl of biotin conjugated antibody for 45 min at 22—24 °C. After washing the assay plate, 100 µl of Streptavidin–Alkaline Phosphatase was added and incubated for 30 min at 22–24 °C. Later the assay plate was washed with PBST and incubated with 100 µl of substrate for 30–45 min at 37 °C. The absorbance of the plate was read at 630 nm to extrapolate concentrations of test samples using the standard curve.

#### Single chain precursor

Proinsulin or single chain precursor (SCP) is part of the impurity associated with the final product. The SCP analysis was performed using ELISA using Human C-Peptide ELISA Kit (Make: R&D Systems Cat No: DICP00). The Quantikine Human Insulin C-Peptide Immunoassay is a 2.5 h solid-phase ELISA designed to measure human Insulin C-Peptide in cell culture supernatant, serum, plasma, and urine^42^.

#### Residual solvent analysis

The organic solvent used during the manufacturing process of insulin aspart are not desirable in the drug substance. The process design ensures that traces of organic material in the drug substance in within the permissible limits. The residual solvent analysis was performed using head space gas chromatography. The recommended limits for particular organic solvent and the method details are documented in pharmacopoeia^43^.

#### Biosimilarity limits

A quality range approach was used to establish the biosimilarity between BGL-ASP and reference insulin aspart. The quality range limits were determined by analyzing 12 reference product batches and expressing limits as mean ± 3 standard deviation. The values for BGL-ASP batches were expected to fall within the established limits to confirm the similarity^44^.

## Results

The characterization study focuses on identifying differences between BGL-ASP and reference insulin aspart using state-of-the-art analytical techniques related to structural and physicochemical analysis. The batches used for clinical trials of BGL-ASP were utilized in the characterization studies and hence considered as representative batches of the manufacturing process.

### Higher order structural analysis

A native and stable higher-order structure of a biotherapeutic is essential for *in-vivo* efficiency and safety of the molecule. Biotherapeutics are large molecules with complex three-dimensional structure. The correct three-dimensional structure of the molecule is a critical requirement for the functionality and stability of the molecule. The disulphide linkage and hydrogen bonds predominantly dictate the native conformation of the molecule. The conformation of the molecule might be affected by the production and purification process and hence establishing conformational similarity is a critical requirement. In this study, the primary structure analysis for BGL-ASP was determined using LC–MS and LC–MS/MS methodology. The far-UV CD study (190–250 nm region) was used to predict the relative percentage of secondary structural element such as α-helix, β-sheet and random coil. The near-UV CD and intrinsic fluorescence data provides an insight into the tertiary structure of insulin aspart. The secondary structure for BGL-ASP was determined using FTIR based analysis and the tertiary/ quaternary structure was determined using DSC based analysis. Higher order structural analysis was also aided by NMR based analysis of BGL-ASP.

The excipients present in the formulation such as phenol and metacresol absorb in the UV region and tend to interfere with the spectroscopic analysis. Hence for CD, Fluorescence and NMR spectroscopy, the drug substance was extracted from the drug product. For FTIR spectroscopy and DSC techniques, the drug product was used to identify the structural conformation.

#### Mass spectrometry analysis of insulin aspart

The LC–MS based analysis demonstrates that the molecular weight of intact (as shown in Table 5) and reduced insulin aspart (as shown in Table 6) was highly similar for BGL-ASP and reference product. The reduction of insulin aspart results in the formation of A-chain and B-chain and the theoretical molecular weights for the peptides were compared with data obtained for BGL-ASP and reference product.

**Table 5:** Intact mass data of insulin aspart.

Product details	Theoretical mass of insulin aspart (Da)	Generated masses (Da)
Reference insulin aspart	5825.64	5825.51(± 1 Da)
BGL-ASP		5825.52 (± 1 Da)

**Table 6:** Reduced mass data of insulin aspart.

Product details	A-Chain		B-Chain	
	Theoretical mass (Da)	Generated mass (Da)	Theoretical mass (Da)	Generated Mass (Da)
Reference insulin aspart	2382.71	2382.86 (± 1 Da)	3447.93	3447.30 (± 1 Da)
BGL-ASP		2382.86 (± 1 Da)		3447.31 (± 1 Da)

The intact molecular mass of BGL-ASP was found to be highly similar to reference insulin aspart.

The molecular mass for A-chain and B-chain of BGL-ASP was found to be highly similar to reference insulin aspart.

#### Peptide mass fingerprinting under reducing and non-reducing conditions

**Figure 3:**
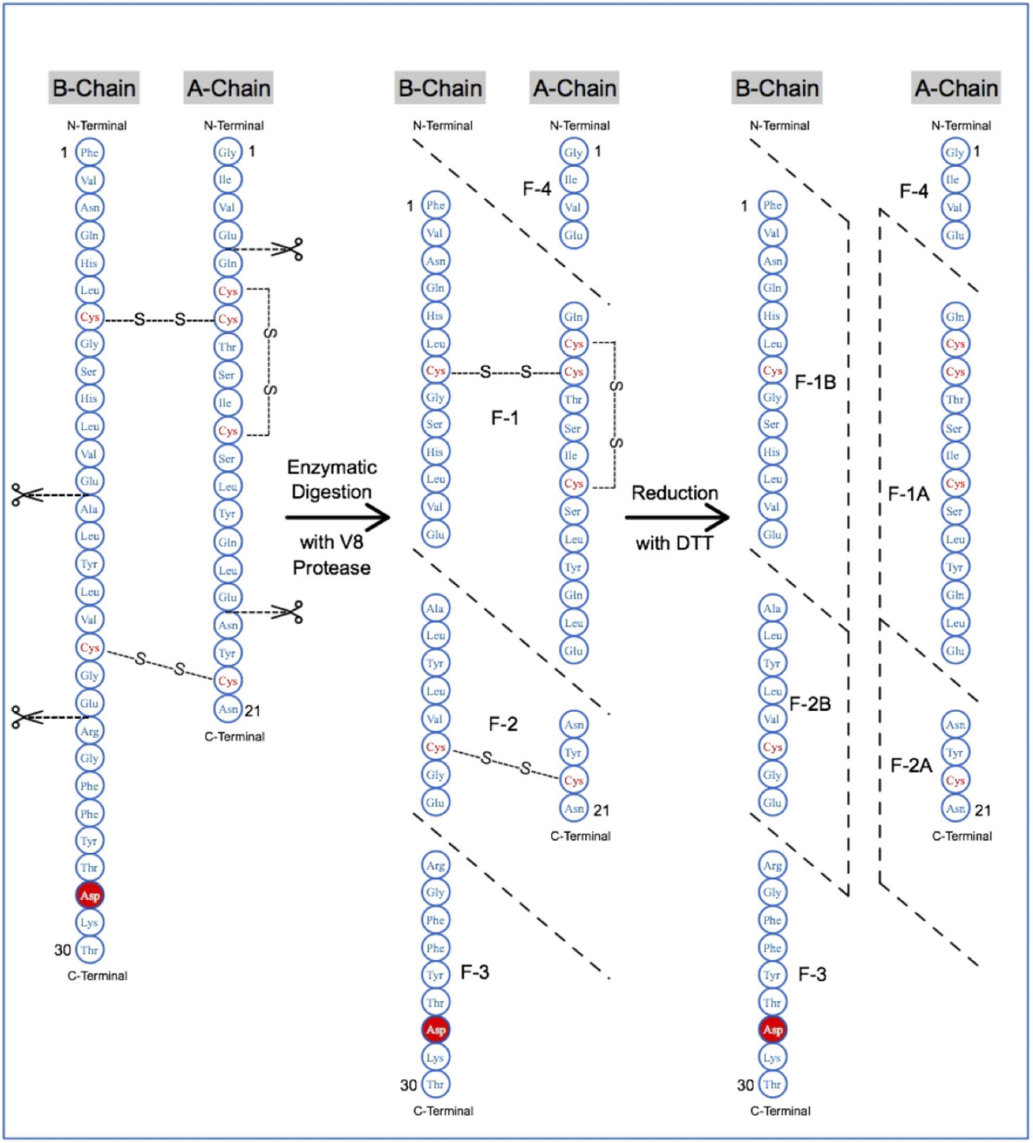
Potential V8 protease cut sites for peptide mapping of non-reduced and reduced insulin aspart. Insulin aspart consists of three disulphide linkages and the pattern of fragments formed under reducing and non-reducing conditions assisted in concluding the presence of native disulphide bonds.

The *Staphylococcus* strain V8 protease specifically cleaves peptide bonds on the carboxyl side of aspartic and glutamic acid residues and was utilized in peptide mapping of insulin aspart to demonstrate the presence of native disulphide bonds. The Fig. 3 demonstrates the structure of insulin aspart with the potential cut sites and the fragments generated pre and post reduction reaction with dithiothreitol (DTT). Comparison of the RP-HPLC profile of the peptide mixture for BGL-ASP and reference product and sequencing of the peptides formed assisted in demonstrating the presence of native disulphide bonds and confirmation of the amino acid sequence.

The Fig. 3 demonstrates the structure of insulin aspart held intact by the presence of two interchain and an intrachain disulphide bond. The enzymatic digestion with V8 protease resulted in the formation of fragments/peptides such as F1, F2, F3 and F4. The fragments F1 and F2 are held together by the presence of the interchain disulphide bonds. The reduction of the disulphide bonds resulted in the formation of additional fragments F1A, F1B, F2A and F2B. Based on the generation of a particular set of peptides under non-reducing and reducing conditions, the presence of native disulphide linkages was confirmed.

##### RP-HPLC profile of V8 enzyme digested Insulin Aspart

**Figure 4:**
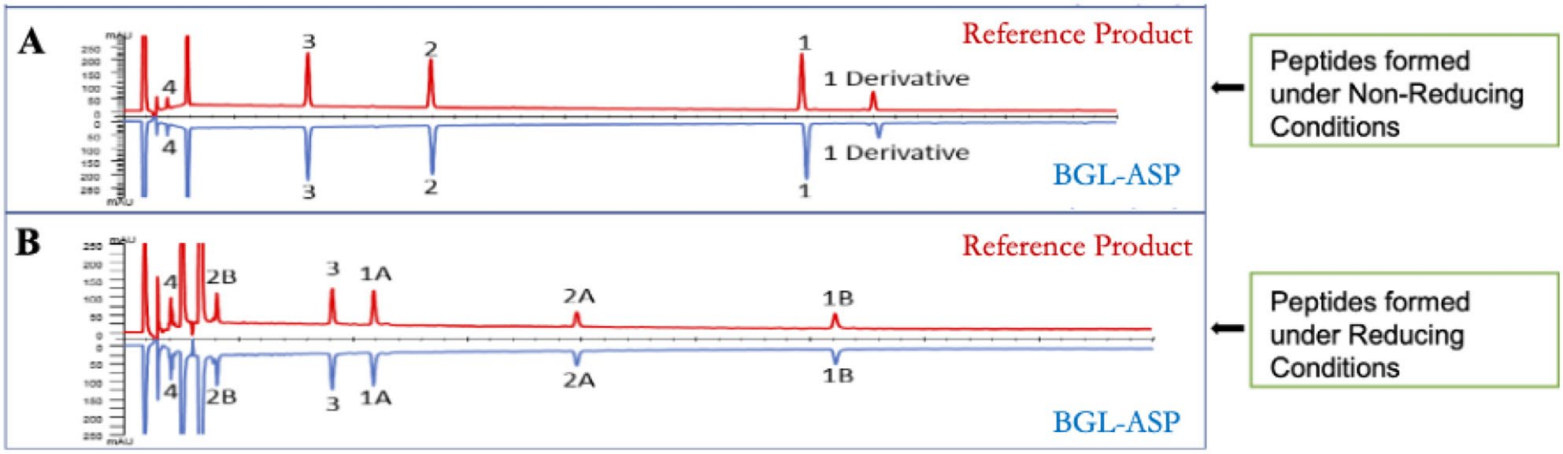
Comparative RP-HPLC based analysis of peptide mixture of BGL-ASP and reference product under reducing and non-reducing conditions. The analysis assisted in confirming the presence of native disulphide linkage in BGL-ASP batches. The panel A and B represents mirror images of chromatograms of V8-generated peptides of BGL-ASP and reference product under non-reducing and reducing conditions, respectively.

The Fig. 4 demonstrates a comparative RP-HPLC profile of the peptide mixture generated post V8 enzymatic digestion of BGL-ASP and reference product. The profile shows high similarity in peak intensities and retention times. The trace peaks were also comparable and no new peaks were apparent in BGL-ASP chromatograms, on comparison with the reference product.

The Fig. 4A and B, demonstrates a comparative RP-HPLC profile for peptides formed post V8 enzymatic digestion for BGL-ASP and reference insulin aspart, under non-reducing and reducing conditions, respectively. The chromatograms were found to be highly similar and the generated mass of the fragments determined using LC–MS analysis (shown in Tables 7 and 8) were found to be identical to the theoretical mass, thus confirming the presence of the native disulphide linkage and similarity to reference product. The RP-HPLC retention times for the generated fragments under non-reducing and reducing conditions were also found to be similar.

**Table 7:** Amino acid sequence and retention time profile of peptide fragments formed under non-reducing conditions.

Peptide details	Amino acid composition	Theoretical mass (Da)	Generated mass (Da)	Retention time of the peptides (min)
Fragment 4	GIVE	416.22	416.22 (± 1 Da)	2.0 ± 0.1
Fragment 1	QCCTSICSLYQLEFVNQHLC GSHLVE	2967.30	2968.23 (± 1 Da)	31.0 ± 0.1
Fragment 2	NYCNALYLVC GE	1376.57	1376.57 (± 1 Da)	14.0 ± 0.1
Fragment 3	RGFFYTDKT	1134.20	1133.55 (± 1 Da)	8.3 ± 0.1

**Table 8:** Amino acid sequence and retention time profile of peptide fragments formed under reducing conditions.

Peptide details	Amino acid composition	Theoretical mass (Da)	Generated mass (Da)	Retention time of the peptides (min)
Fragment 4	GIVE	416.22	416.22 (± 1 Da)	2.3 ± 0.1
Fragment 1B	QCCTSICSLYQLE	1490.70	1488.84 (± 1 Da)	12.6 ± 0.1
Fragment 2A	NYCN	494.50	498.17 (± 1 Da)	20.1 ± 0.1
Fragment 3	RGFFYTDKT	1134.20	1133.58 (± 1 Da)	10.6 ± 0.1
Fragment 2B	ALYLVCGE	867.00	865.43 (± 1 Da)	4.0 ± 0.1
Fragment 1A	FVNQHLCGSHLVE	1481.60	1480.79 (± 1 Da)	31.3 ± 0.1

The Table 8 demonstrates the RP-HPLC retention times for the fragments generated under reducing conditions.

The fragments shown in Table 8 were subjected to LC–MS/MS analysis and it was observed that the amino acid sequence of BGL-ASP and reference product are highly similar, thus confirming the primary structure of the molecule.

#### UV circular dichroism spectroscopy

**Figure 5:**
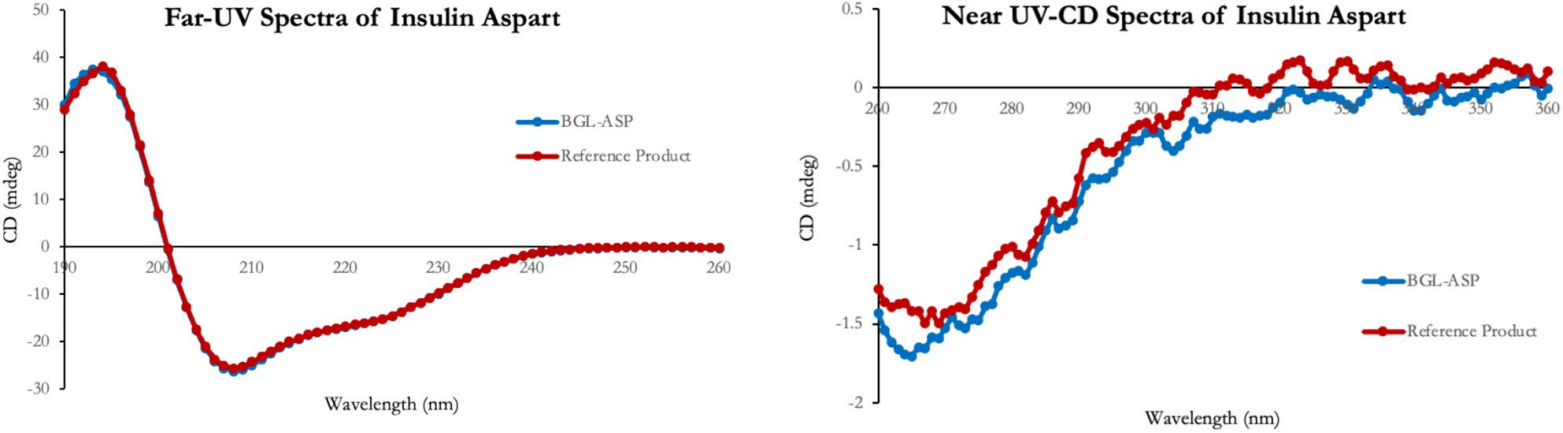
Comparative far-UV and near-UV CD spectra of reference insulin aspart and BGL-ASP. The far-UV and near UV-CD spectra of BGL-ASP & reference insulin aspart were found to be highly similar.

The comparative far and near-UV CD spectra for BGL-ASP and reference product is shown in Fig. 5. The far-UV CD spectra of BGL-ASP and reference marketed insulin aspart were found to be highly similar with characteristics spectra of an alpha helix with positive maxima at 193 nm and negative maxima at 208 nm and 222 nm^45^. The near-UV CD spectra of insulin aspart were generated to demonstrate the equivalence of tertiary structures.

Table 9 represents the generated data set from secondary structural characterization of insulin aspart samples by means of far-UV CD spectroscopy.

**Table 9:** Analytical data of secondary structural elements for insulin aspart.

Product analyzed	Secondary structural elements (%)				
	αhelix	βsheets	β turn	Random coil	Total
Reference product	26.3	51.8	0	22	100
BGL-ASP	26	53	0	21	100

The generated far UV CD data confirmed the presence of highly similar α-helix, β-sheets and random coil content for reference product and BGL-ASP.

#### NMR spectroscopy

NMR Spectroscopy is one of the most powerful techniques to decipher the structural information of biomolecules in solution at atomic resolution. Magnetically active nuclei like ^1^H, ^15^N, and ^13^C behave like a small spinning magnets, and when kept under the influence of an external magnetic field, will precess about the applied magnetic field. The frequency of precession depends upon chemical environment of the atom and gives rise to a particular resonance frequency, known as the Larmor frequency (ω). This frequency also depends on the applied magnetic field. Thus, in order to have uniform scale across different magnetic fields under which NMR data can be measured, the precession frequency is expressed as chemical shift (δ). In case of a protein and peptide, each amino acid is not only influenced by neighbouring amino acids in the polypeptide chain but also by amino acids that come close due to the three-dimensional arrangement in the tertiary structure. This leads to a different local environment for each atom in the protein and hence a different chemical shift.

The chemical shift of the nucleus is extremely sensitive to chemical environment and any subtle changes caused by changes in conformation, aggregation, and/or changes in buffer conditions will manifest as changes in the chemical shifts observed in the NMR spectra. Hence, NMR signals in the signature regions of 1D and 2D NMR spectra act as a fingerprint of the molecule in a particular condition. These signature regions can be used to compare the conformation of peptide molecule in different formulations and assist in establishing the structural similarity. Proton 1D spectrum and a range of 2D NMR spectra can be used as independent methods for assessing structural similarity. The 2D-COSY (homonuclear correlation spectroscopy), 2D-TOCSY (Total Correlation Spectroscopy) and 2D-^1^H,^13^C-HSQC (heteronuclear single quantum coherence) experiments provide through-bond correlations. NOESY (Nuclear Overhauser Effect Spectroscopy) experiments provide information of through-space correlation among atoms. The NOESY patterns are used to identify secondary structural features such as α-helices and β-strands for certain regions of the sequence. Similarly, secondary ^13^C chemical shifts from ^1^H,^13^C-HSQC provide secondary structure information as a function of the amino acid sequence. The NMR chemical shifts are highly sensitive to changes in the chemical environment of the nucleus and identical spectra of samples unequivocally confirm the identical structure.

##### Proton 1D spectra of BGL-ASP and reference product

**Figure 6:**
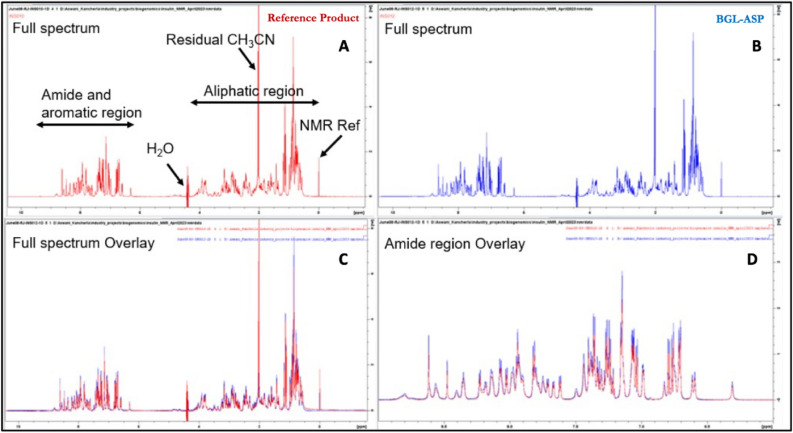
Comparative Proton 1D spectra of BGL-ASP and reference product. (**A**): Full 1H 1D spectrum of reference product along with annotation of signals and spectral regions. (**B**): Full spectrum of BGL-ASP. (**C**): Overlay of the full 1H 1D spectra of both the samples. (**D**): Overlay of the amide proton region of the 1H 1D spectrum. The spectra were identical with respect to spectral dispersion, linewidth and signal intensity. The recorded observation strongly suggested high structural similarity between BGL-ASP and reference product.

The overlay of 1D NMR spectrum for both the samples demonstrated the presence of the hydrogen peak with same integral value, with same line width at definite ppm value. Down fielded signals above 9 ppm and up-fielded signals below 0 ppm, which are indicative of a well folded polypeptide conformation were not observed in the 1D spectrum. Additionally, the spectral dispersion observed in the 1D ^1^H spectrum of both the samples indicated that the peptide is likely to be present either in random coil or a helical conformation. The overlay spectra (Fig. 6) revealed that both the spectra were identical and hence indicated the structural similarity of BGL-ASP and reference product.

##### 2D-^1^H,^1^H-COSY spectra of BGL-ASP and reference product

**Figure 7:**
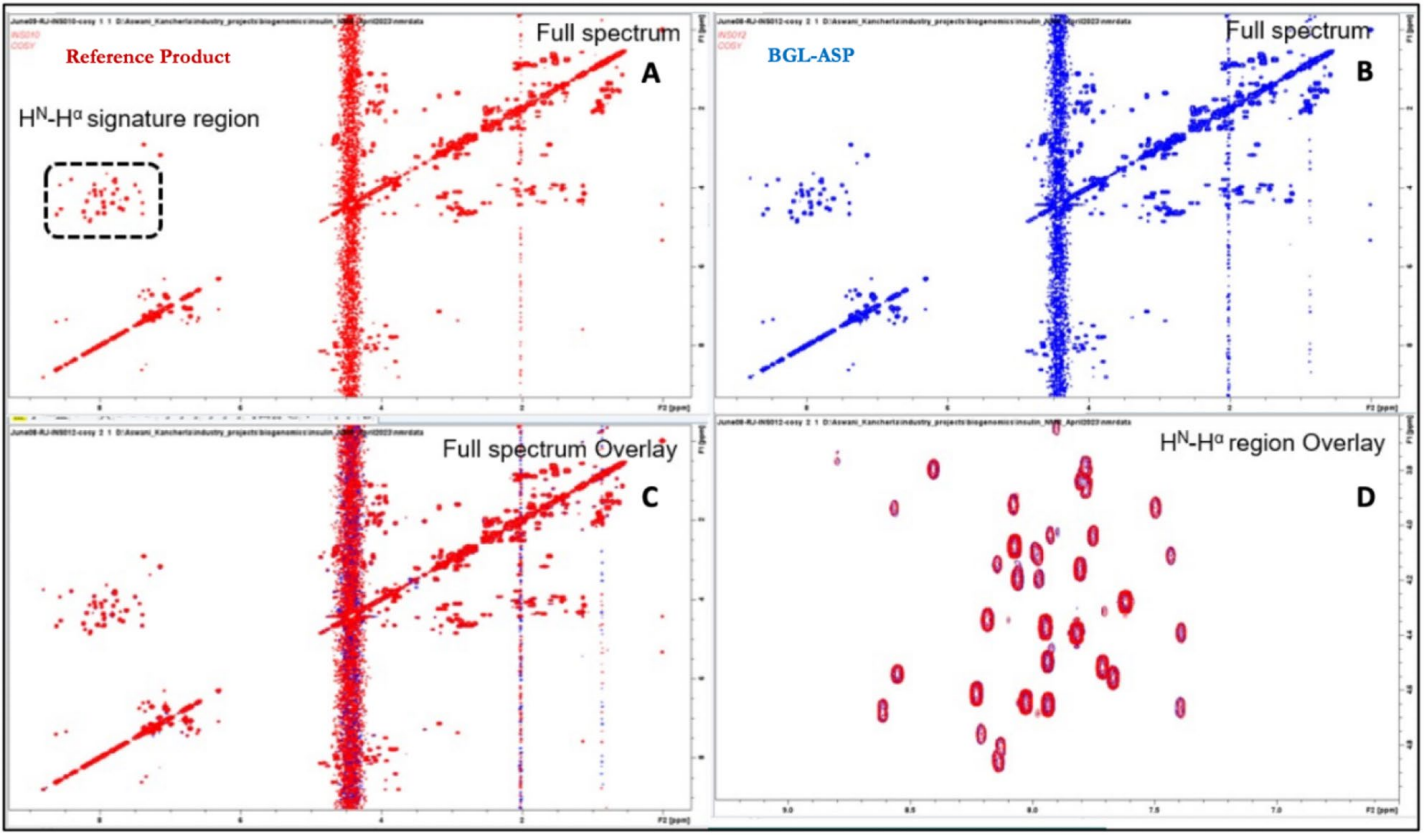
Comparative COSY spectra of BGL-ASP and reference product. (**A**): Full 2D cosy spectrum of reference product. The H^N^H^α^ signature region is highlighted by a dashed rectangle. (**B**): Full 2D cosy spectrum of BGL-ASP. (**C**): Overlay of the full 2D COSY spectra of reference product and BGL-ASP. (**D)**: Overlay of the H^N^H^α^ signature region of the 2D COSY spectra of reference product and BGL-ASP. The H^N^-H^α^ signature region of the COSY spectrum is a fingerprint of the molecule in a given condition. Similarity of the spectra confirmed the structural similarity of BGL-ASP and reference product.

The 2D COSY spectrum provides spectral correlations for protons that are coupled via 3 or less than 3 covalent bonds. In the 2D-^1^H,^1^H COSY, spectra the peak observed diagonally corresponds to the signals observed in 1D NMR spectra, whilst positions of cross peaks observed in the COSY corresponds to pairs of protons or equivalent groups of protons which are coupled by 3 or less than 3 covalent bonds. The H^N^-H^α^ signature region consists of correlations between backbone amide proton H^N^ and alpha proton H^α^ and hence has one signal per amino acid except for proline which lacks the backbone amide proton. The number of cross peaks observed in H^N^-H^α^ signature region of COSY spectrum for both the samples was 42. The expected number of correlations in this region was about 52. The missing peaks could be overlapped or could be broadened due to conformational exchange. The overlay 2D-^1^H,^1^H COSY spectra (Fig. 7) showed that, the position of diagonal peaks and position of cross peaks for both samples are exactly overlapped with each other and that the number of peaks observed in H^N^-H^α^ signature region of the COSY spectrum were identical. These observations strongly supported the structural similarity of BGL-ASP and reference product.

##### 2D-^1^H,^1^H-TOCSY spectra of BGL-ASP and reference product

**Figure 8:**
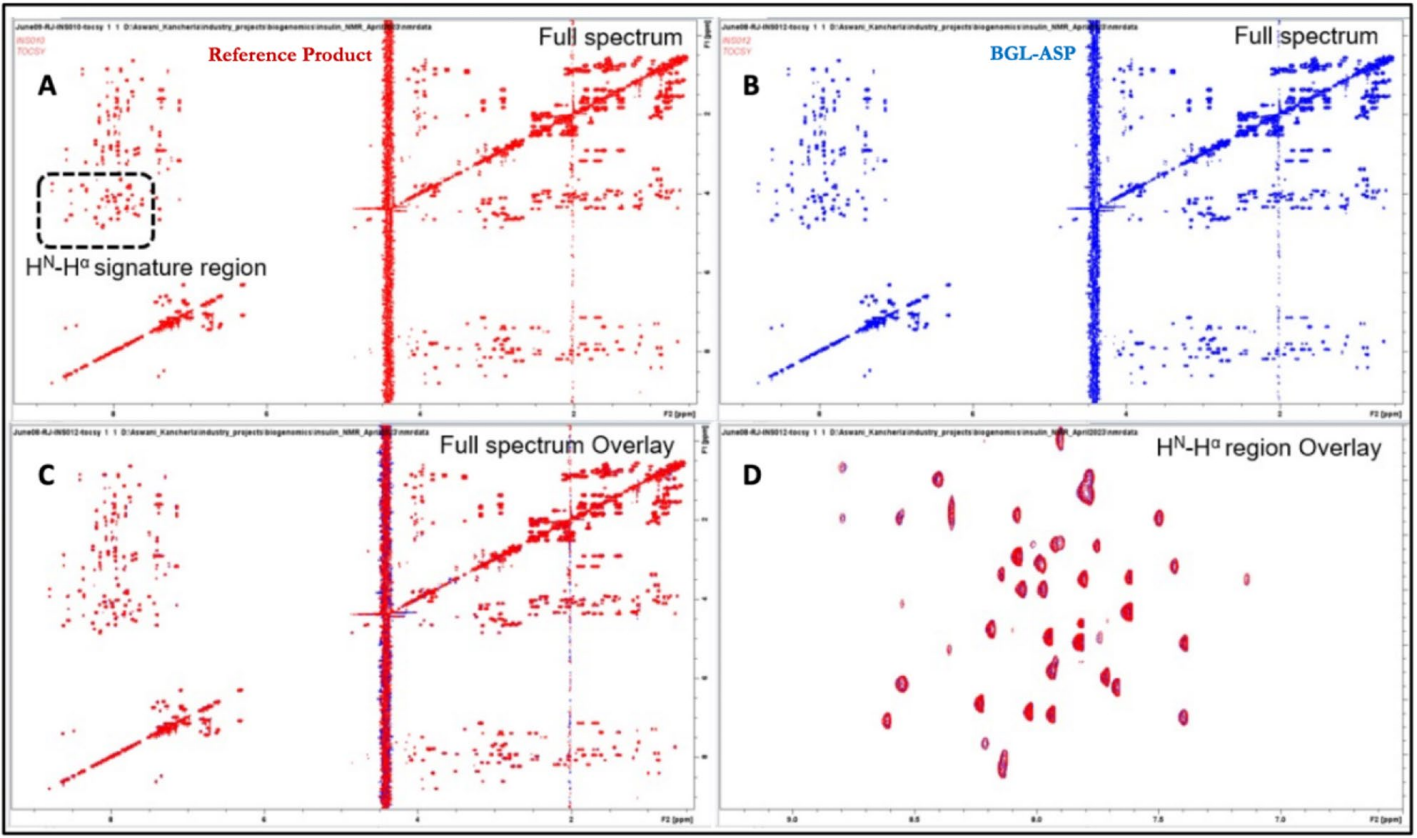
Comparative TOCSY spectra of BGL-ASP and reference product. (**A**): Full 2D TOCSY spectrum of reference product. The H^N^H^α^ signature region is highlighted by a dashed rectangle. (**B**): Full 2D TOCSY spectrum of BGL-ASP. (**C**): Overlay of the full 2D TOCSY spectra of reference product and BGL-ASP. (**D**): Overlay of the H^N^H^α^ signature region of the 2D TOCSY spectra of reference product and BGL-ASP. Similarity of the spectra confirmed the structural similarity of BGL-ASP and reference product.

In the 2D-^1^H,^1^H TOCSY spectrum, the peaks observed diagonally correspond to the signals observed in 1D 1H NMR spectrum, whilst positions of cross peaks observed correspond to the side chain protons of the respective aromatic and aliphatic amino acids which are in a network of coupled spins. Thus, the TOCSY spectrum provides correlations between all protons which are part of a coupled network at every proton and enables easy identification of types of amino acids present in the molecules. The H^N^-H^α^ signature region of the TOCSY spectrum is similar to that of COSY spectrum except that the correlations between H^N^ and H^β^ of Serine and Threonine residues also show up in this region. The overlay of ^1^H,^1^H TOCSY spectra (Fig. 8) for both the samples showed that the position of diagonal peaks and cross peaks match exactly with each other. Thus, the TOCSY spectra strongly suggested the structural similarity of BGL-ASP and reference product.

##### 2D-^1^H,^1^H-NOESY spectra of BGL-ASP and reference product

**Figure 9:**
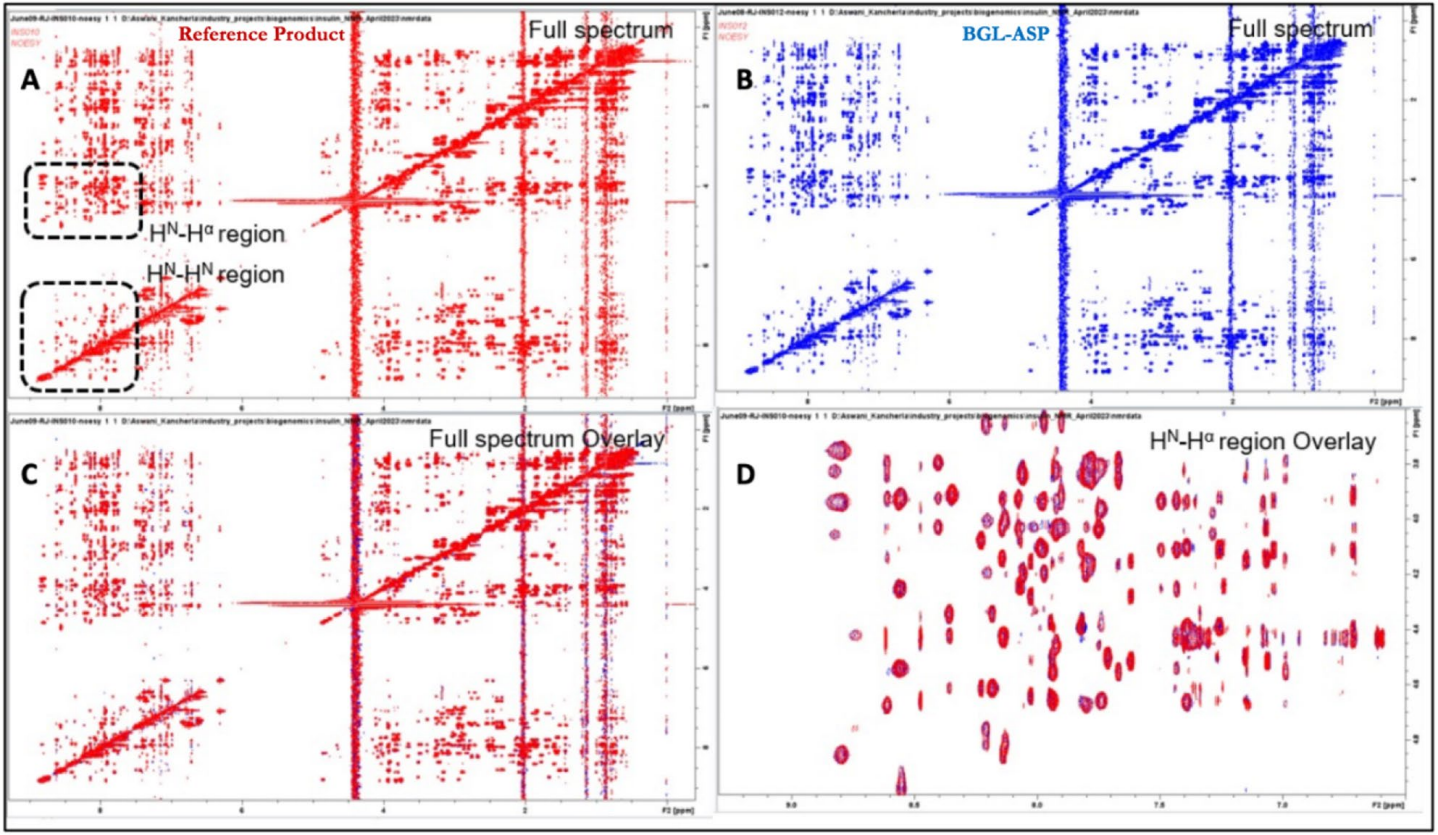
Comparative NOESY spectra of BGL-ASP and reference product. (**A**): Full 2D NOESY spectrum of reference product. The H^N^H^α^ and H^N^H^N^ signature regions are highlighted by dashed rectangles. (**B**): Full 2D NOESY spectrum of BGL-ASP. (**C**): Overlay of the full 2D NOESY spectra of reference product and BGL-ASP. (**D**): Overlay of the H^N^H^α^ signature region of the 2D NOESY spectra of reference product and BGL-ASP. Similarity in the NOESY correlations in the H^N^-H^α^ and H^N^-H^N^ regions indicates the similarity of the secondary and tertiary structures exhibited by reference product and BGL-ASP.

In the NOESY spectrum, positions of cross peaks observed are due to spin coupling resulting from interactions of spins via space. Hence, a NOESY correlation between 2 protons indicates that the pair of protons are close to each other in space, usually < 6 Ǻ. Folding of protein generates secondary and tertiary structure and, in the process, brings different groups of protons close to each other, resulting in different NOESY patterns. The overlay of NOESY spectra (Fig. 9) for the both the samples showed the same peak position in the entire spectra. Additionally, similarity in the NOESY correlations in the H^N^-H^α^ and H^N^-H^N^ regions indicated the similarity of the secondary structure and tertiary structure exhibited by the BGL-ASP and reference product. Similarity in the NOESY spectra confirmed the presence of identical disulphide linkages for BGL-ASP and reference product.

##### 2D-^1^H,^13^C-HSQC spectra of BGL-ASP and reference product

**Figure 10:**
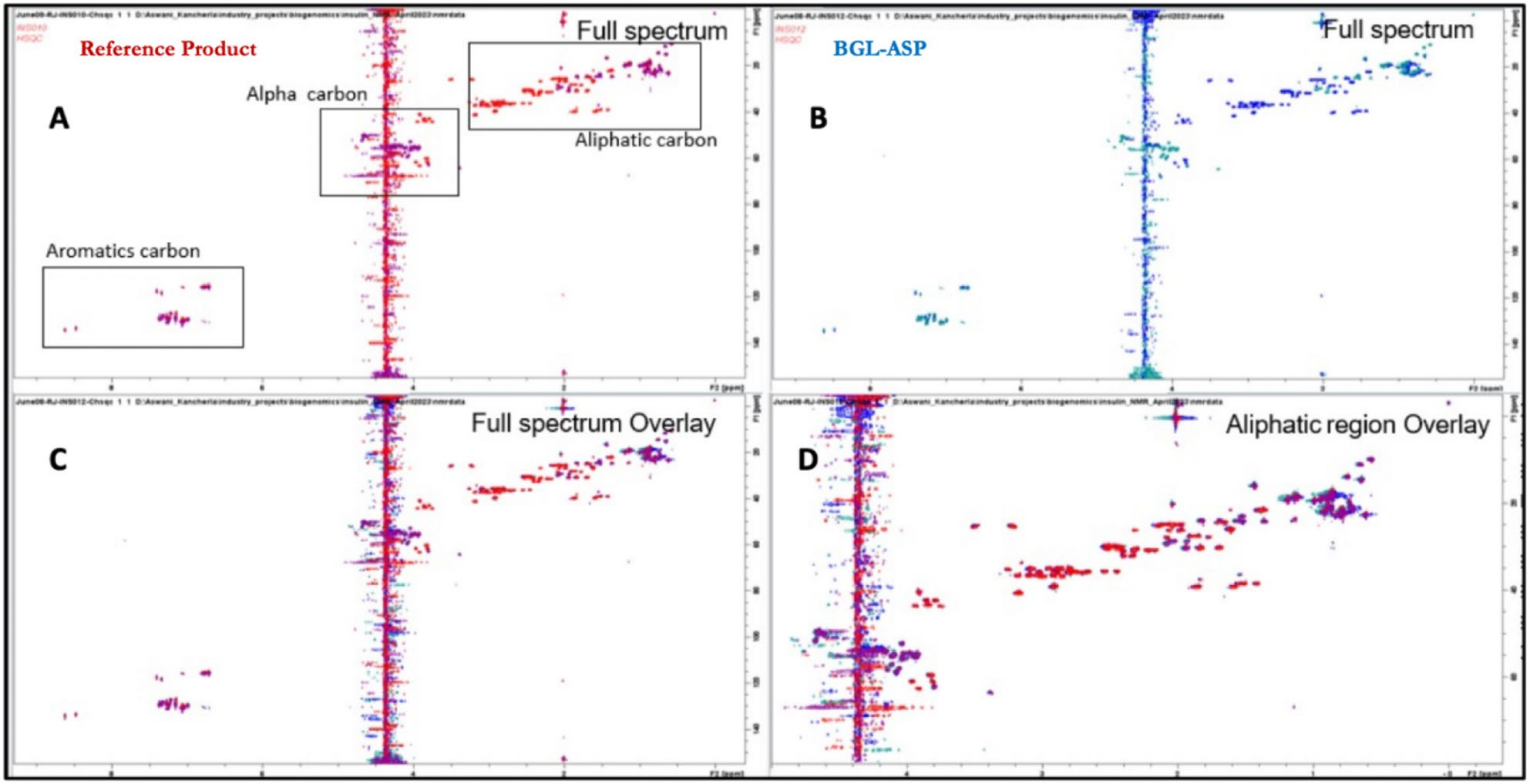
Comparative HSQC spectra of BGL-ASP and reference product. (**A**): Full 2D ^1^H,^13^C-HSQC spectrum of reference product (shown in red & purple). The aromatic carbon, alpha carbon and aliphatic side chain carbon regions are highlighted by rectangles. (**B**): Full 2D ^1^H,^13^C-HSQC spectrum of BGL-ASP (shown in blue and cyan). (**C**): Overlay of the full 2D ^1^H,^13^C-HSQC spectra of reference product and BGL-ASP. (**D**): Overlay of the aliphatic region of the 2D ^1^H,^13^C-HSQC spectra of reference product and BGL-ASP.

The 2D ^1^H,^13^C-HSQC spectrum provides correlations between protons and the directly attached carbons. Carbon chemical shifts are sensitive to the conformation of the molecule and hence similarity of signals in the ^1^H^13^C-HSQC spectrum strongly suggests similarity of the conformation of the molecule. The overlay of ^1^H,^13^C-HSQC spectra (Fig. 10) revealed that the carbon chemical shift values for aromatic carbon, alpha carbon and aliphatic carbons of BGL-ASP and reference product were identical which confirmed the conformational similarity of both the molecules.

The different NMR methodologies confirmed that atom to atom spatial positioning was identical for BGL-ASP and the reference product.

#### FTIR Spectroscopy

**Figure 11:**
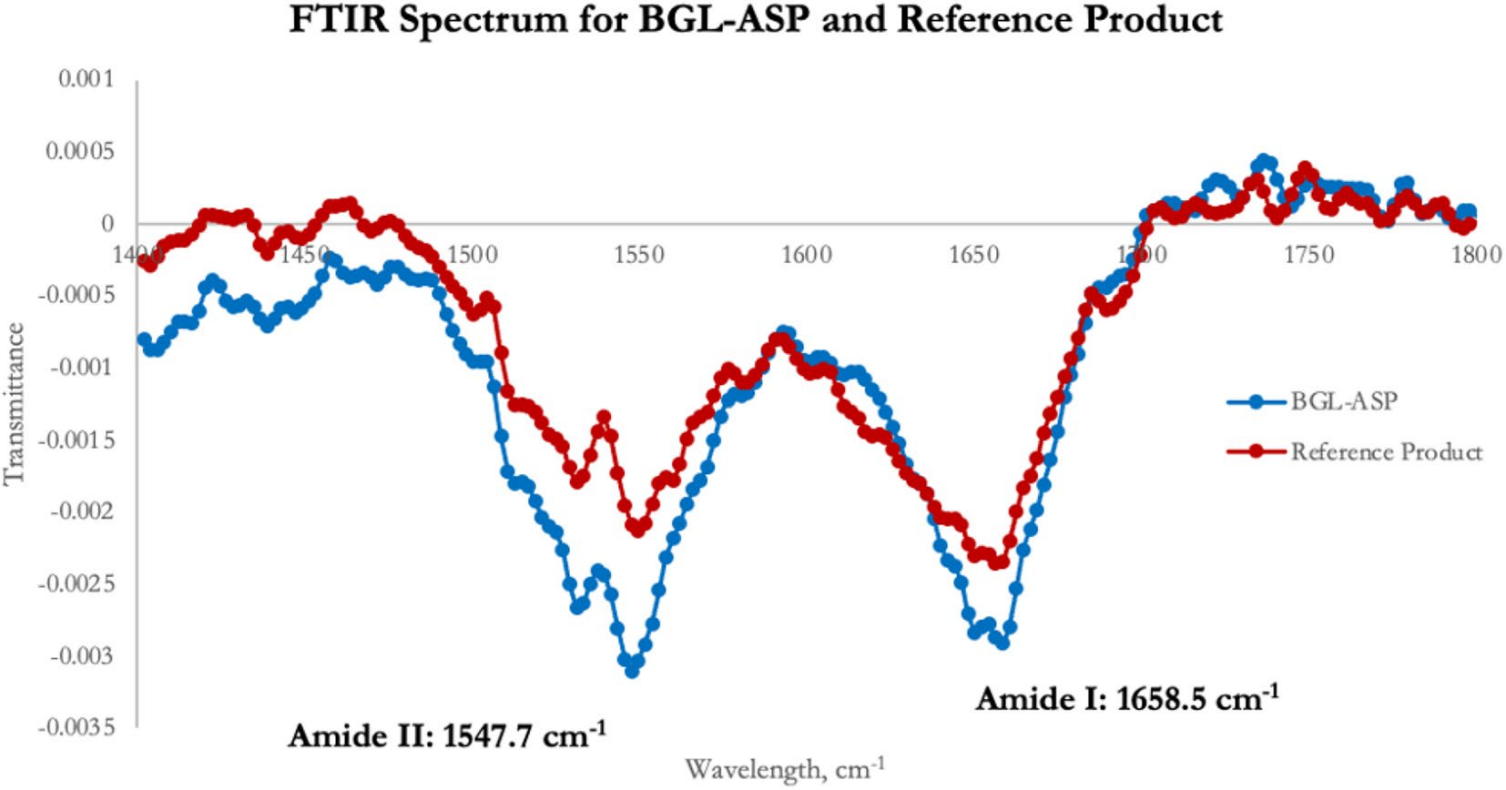
Comparative FTIR spectra of BGL-ASP and reference product. The position of amide I and amide II bands was identified at 1658.5 cm^−1^ and 1547.7 cm^−1^, respectively for both the samples. The FTIR spectra was found to be highly similar in terms of shape and the position of the amide bonds for BGL-ASP and reference product.

FTIR spectroscopy is utilized to study the secondary structure of peptides and proteins as it probes the amide (peptide) bonds responsible for the distinct infrared (IR) signals for the differently folded peptides and proteins. The Amide I, II and III are most commonly used IR spectral regions used for protein structure–function analysis. The FTIR spectrum between 1700 and 1500 cm^−1^ consists of the amide I and II bands resulting from the absorption of the carbonyl amide present in the peptide bond. The shape of these bands are analysed for the determination of the secondary structure content^46–48^. The comparative FTIR spectra for BGL-ASP and reference product is shown in Fig. 11.

The amide I band is due to carbonyl stretching vibrations of the peptide bonds and are influenced by the secondary structures such as α-helix, β-sheet, etc. The amide II band is attributed to NH_2_ deformation in primary amides and form a mixed vibration of N–H bending and C–N stretching in secondary amides. The position and shape of the amide I and amide II bands was recorded between 1700 and 1500 cm^−1^. The position of amide I and amide II bands were identified at 1658.5 cm^−1^ and 1547.7 cm^−1^, respectively for BGL-ASP and reference product batch. The Pearson’s correlation coefficient was used to compare the FTIR spectra and a value closer to + 1.0 indicates a better correlation. The Pearson’s correlation coefficient for these two spectra was found to be 0.9674, thus confirming the high similarity between BGL-ASP and reference product.

#### Differential scanning calorimetry (DSC)

DSC is a useful tool for monitoring equilibrium thermodynamic stability of biomolecules. The DSC technique is routinely utilized to monitor the thermal stability and folding mechanism of biopharmaceuticals. The formulation process and the excipients such as phenol, m-cresol and zinc are critical formulation components related to the conformation of insulin aspart present in solution. The melting temperature (*T*_m_), identified from the DSC thermogram can be effectively compared for reference product and BGL-ASP to demonstrate similarity in hexamer stability in formulation. The Table 10 shows the melting temperature for each of the three phases of insulin aspart.

**Table 10:** Summary of nanoDSC results.

Phases	Reference product	BGL-ASP
	*T*_*m*_ (˚C)	*T*_*m*_ (˚C)
Phase I	66.86	67.10
Phase II	76.15	77.36
Phase III	80.26	83.07

The *T*_*m*_ measured by DSC can be utilized as a predictive tool for identifying the impact of aggregation and chemical modifications on higher order structural attributes^49–52^. The DSC data demonstrated the presence of three different melting phases and similar *T*_*m*_ for BGL-ASP and reference product, thus confirming the presence of highly similar quaternary structures. The Pearson's correlation coefficient for the two thermograms was found to be 0.9893, thus confirming high similarity between BGL-ASP and reference product.

#### Intrinsic fluorescence spectroscopy

**Figure 12:**
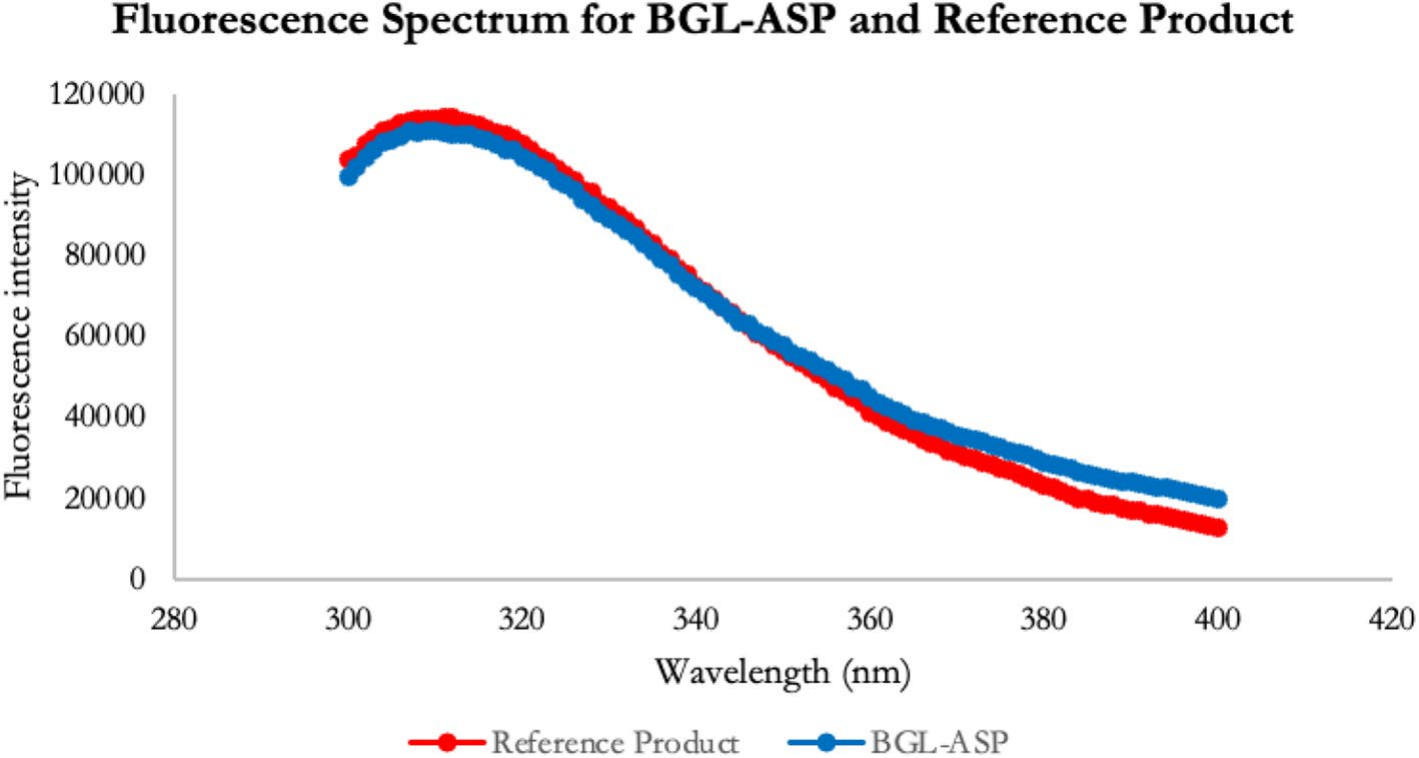
Comparative intrinsic fluorescence spectral profile of reference insulin aspart and BGL-ASP. The blue & red line represents the intrinsic fluorescence spectra of BGL-ASP & reference product, respectively. The emission maxima for both the insulin aspart samples was detected at 310 nm upon excitation at 278 nm.

The intrinsic fluorescence spectroscopy is utilized to study the tertiary structure of insulin aspart. The fluorescence spectra acts as an orthogonal tool to near-UV CD data and further confirms the native conformation of the molecule. The comparative intrinsic fluorescence spectroscopy data for BGL-ASP and reference product is shown in Fig. 12.

The intrinsic fluorescence spectra was found to be highly similar for reference product and BGL-ASP, thus confirming the similarity in tertiary structure.

### Physicochemical characterization

The physicochemical characterization of BGL-ASP involves the use of RP-HPLC based analysis to determine the assay, impurity and excipient content. The higher molecular weight protein (HMWP) content is monitored using SE-HPLC based analysis.

#### RP-HPLC based related impurity analysis of insulin aspart

**Figure 13:**
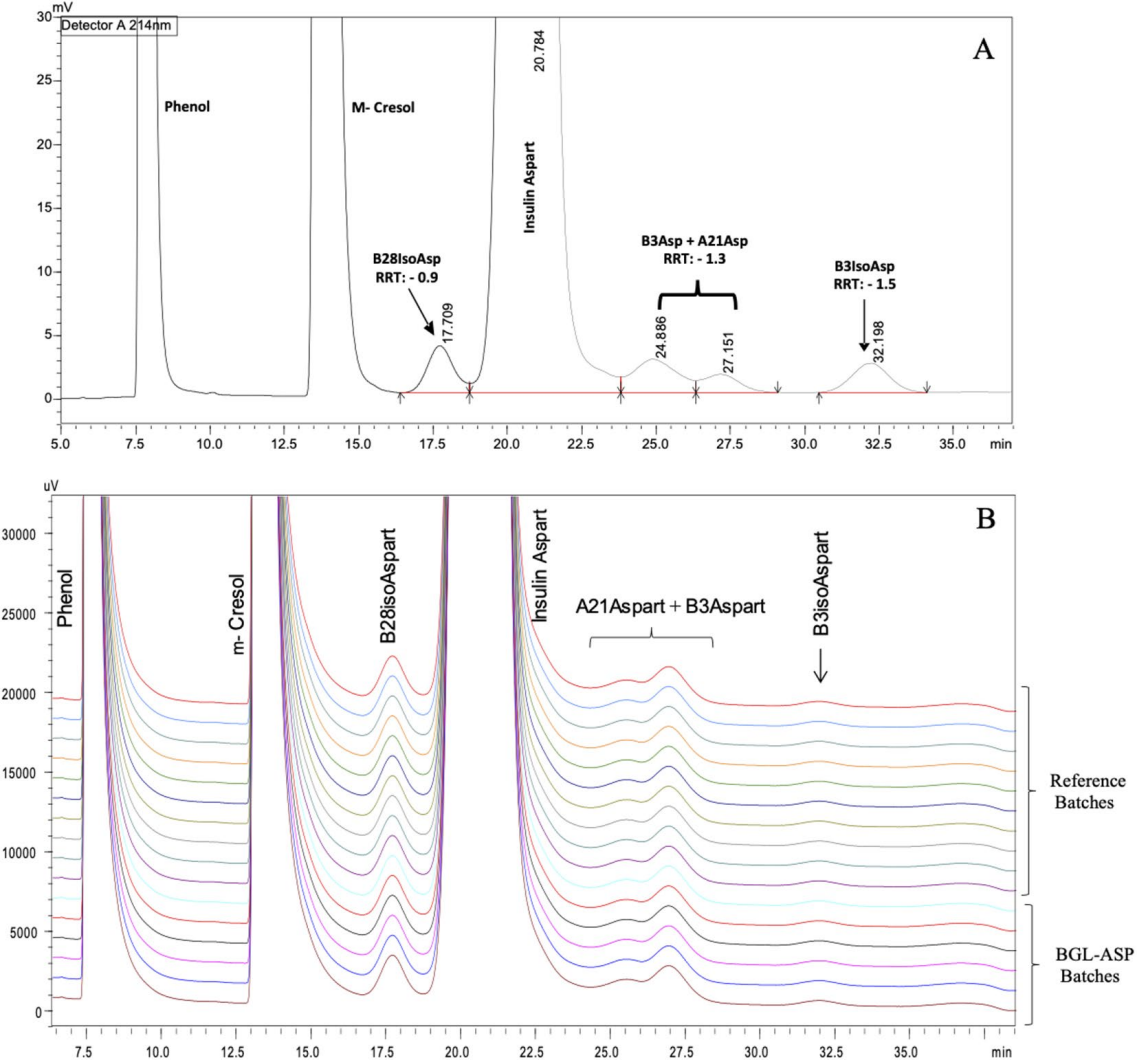
RP-HPLC based separation of related impurities of insulin aspart. The chromatogram in panel A demonstrated the distinct separation of the excipients such as phenol and m-cresol and product related impurities such as B28isoAsp, B3Asp, A21Asp and B3isoAsp. The panel B demonstrates stacked chromatograms for reference and BGL-ASP batches and the profiles were found to be high similar.

The related impurity analysis of insulin aspart was performed using the RP-HPLC technique. The typical impurities formed for insulin aspart are the deamidated (A21Asp) and isomerized forms (B28isoAsp, B3Asp, B3isoAsp) and the RP-HPLC analysis was able to resolve these impurities, thus assisting in quantitative analysis and establishing biosimilarity limits. The RP-HPLC based analysis was also utilized in determining the assay content and the concentration of the preservatives used in insulin aspart formulation such as phenol and m-cresol. Nine batches of BGL-ASP and twelve batches of reference product were tested using RP-HPLC based analysis. The comparative RP-HPLC chromatogram for BGL-ASP and reference product is shown in Fig. 13.

**Figure 14:**
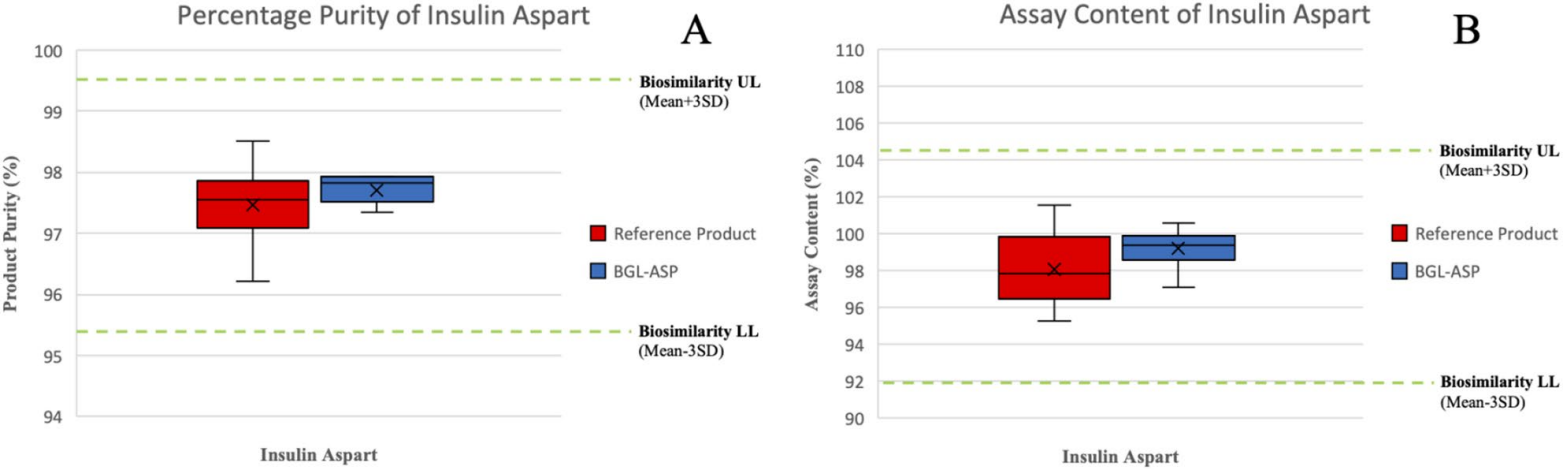
Graphical representation for assay content and purity determined as per RP-HPLC in BGL-ASP batches. The panel A and B demonstrates the percentage purity and assay content of insulin aspart batches, respectively. The green lines demonstrate the upper and lower limit of biosimilarity. The percentage purity and assay content are found to be within the established biosimilarity limits.

The related impurities are marked in the chromatogram and were found to be well resolved. The percentage purity and assay content of reference insulin aspart batches was determined and the biosimilarity limits were calculated using a multiplier of x = 3 (mean ± 3*SD) as a generally appropriate multiplier for the similarity assessment. The biosimilarity limits and the data obtained for percentage purity and assay content for BGL-ASP and reference product is shown in Fig. 14.

The data demonstrates that the percentage purity and assay content of BGL-ASP batches are within the established biosimilarity limits, identified by analyzing multiple batches of reference product.

#### SE-HPLC based HMWP analysis in insulin aspart

**Figure 15:**
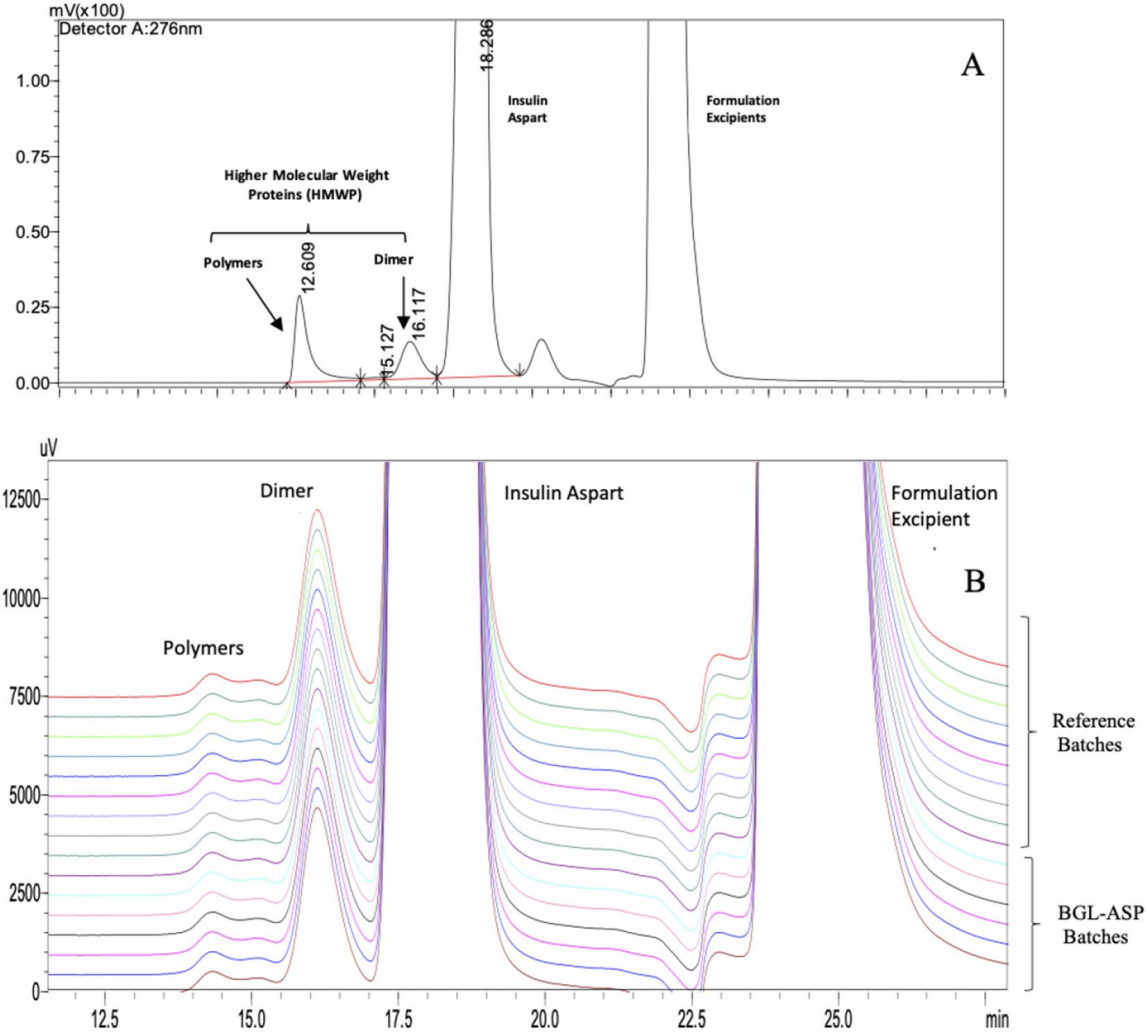
SE-HPLC based separation of HMWP of insulin aspart. The chromatogram in panel A is a representative chromatogram for stressed sample of insulin aspart drug product and demonstrates distinct separation of monomeric, dimeric and polymeric form of insulin aspart. The panel B demonstrates stacked chromatograms for reference and BGL-ASP batches and the profiles were found to be high similar.

The SE-HPLC based analysis is utilized to determine the HMWP content in insulin aspart formulation. The dimeric and multimeric forms of insulin aspart are resolved using SE-HPLC. Nine batches of BGL-ASP and twelve batches of reference product were tested using SE-HPLC based analysis. The comparative SE-HPLC chromatogram for BGL-ASP and reference product is shown in Fig. 15.

**Figure 16:**
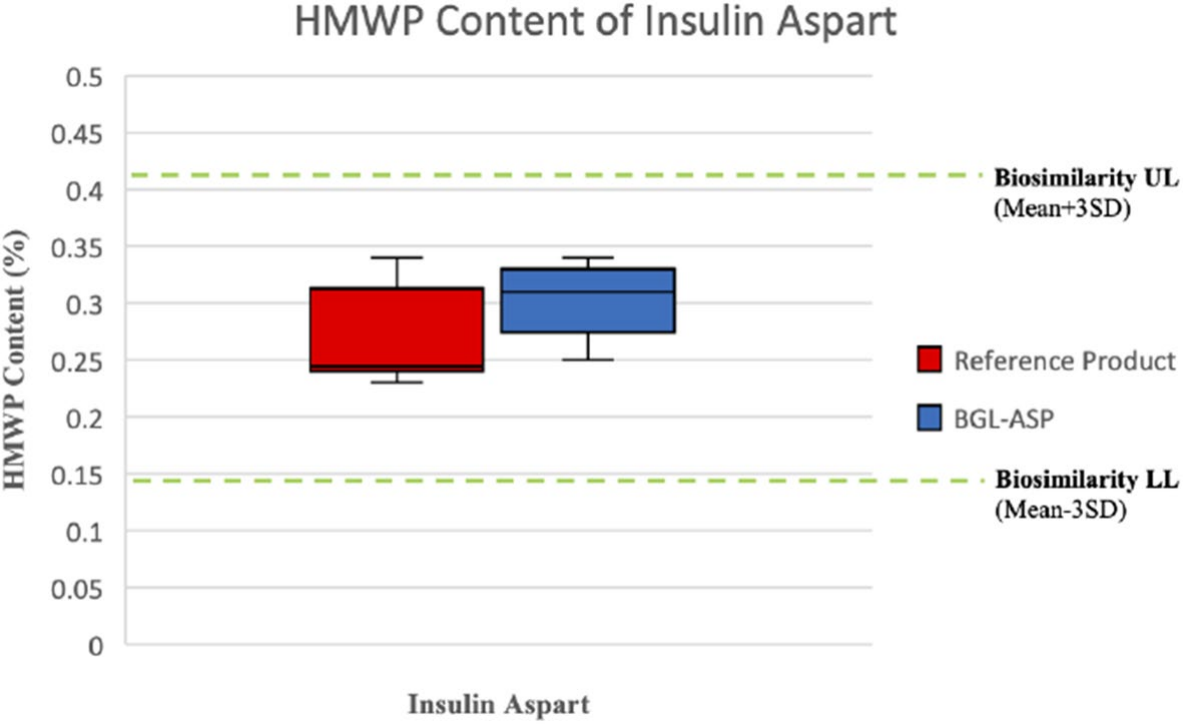
Graphical Representation of HMWP content for BGL-ASP batches and biosimilarity limits. The graph demonstrates that the HMWP content in BGL-ASP batches are within the mean ± 3.0*SD limit.

The HMWP are marked in the chromatogram and were found to be well resolved. The HMWP content in reference product and BGL-ASP batches and the biosimilarity limits are demonstrated in Fig. 16.

The HMWP content in BGL-ASP batches was found to be within the established biosimilarity limits, identified by analysing multiple batches of reference product.

#### Preservative content, pH and zinc content in insulin aspart

RP-HPLC based methodology has been utilized to determine the preservative content such as phenol and m-cresol in insulin aspart formulations. The Table 11 demonstrates the content of the excipient in reference product and BGL-ASP formulation.

**Table 11:** Excipient content in reference product and BGL-ASP batches.

Excipient	Identified range	Minimum–maximum observed range (number of lots tested)	
		Reference product	BGL-ASP
Phenol	1.41–1.61 mg/ml	1.45–1.55 (12)	1.43–1.61 (9)
m-Cresol	1.61–1.85 mg/ml	1.66–1.79 (12)	1.57–1.79 (9)
pH	7.10–7.50	7.27–7.42 (12)	7.20–7.42 (9)

The phenol, m-cresol content and pH were found to be within the identified range for BGL-ASP. The zinc content in insulin aspart was determined using atomic absorption spectroscopy. The zinc content for BGL-ASP and reference product was found to be within the range of 15–25 ug/100 IU.

#### Stability studies

**Figure 17:**
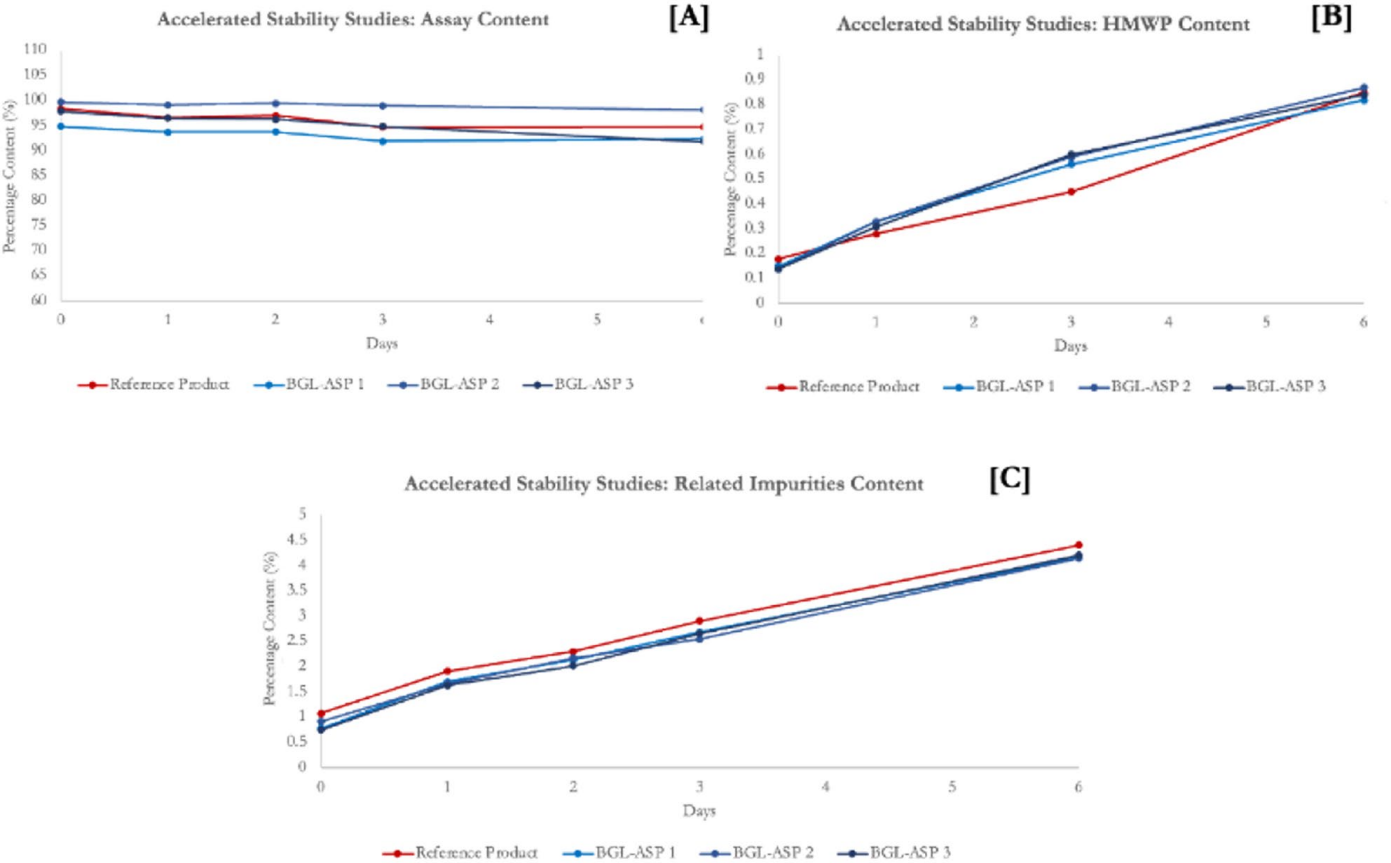
Accelerated stability studies. Graphical representation for comparative accelerated stability study of BGL-ASP and reference product. The panel (**A**)–(**C**) demonstrate the assay, HMWP and related impurity content, respectively. The data is documented for three batches of BGL-ASP and a single batches of reference product.

**Figure 18:**
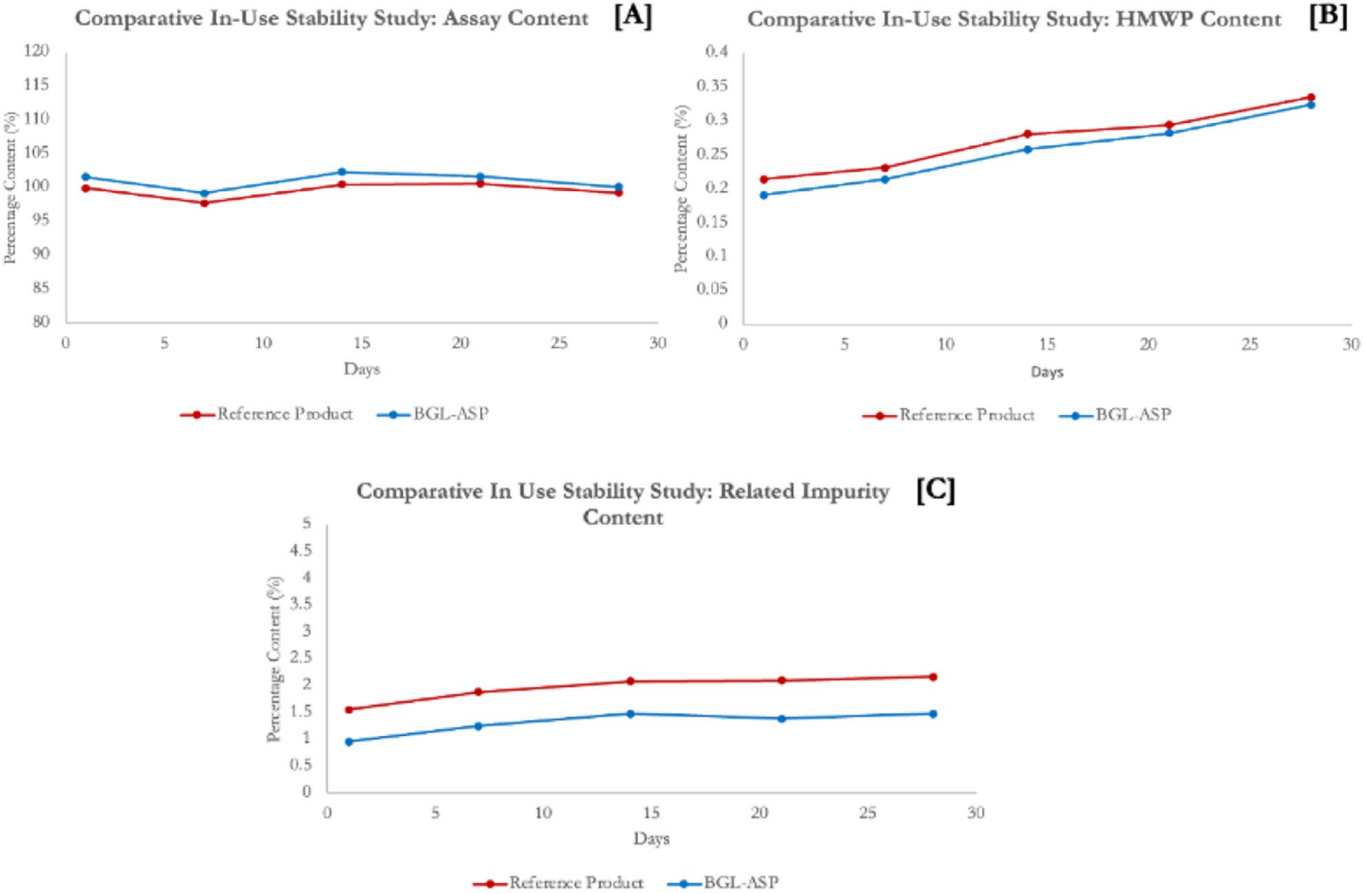
In-Use stability studies. Graphical Representation for in-use stability studies of BGL-ASP. The panel (**A**)–(**C**) demonstrate the assay, HMWP and related impurity content, respectively. The data is documented for a single batch of BGL-ASP and reference product.

**Figure 19:**
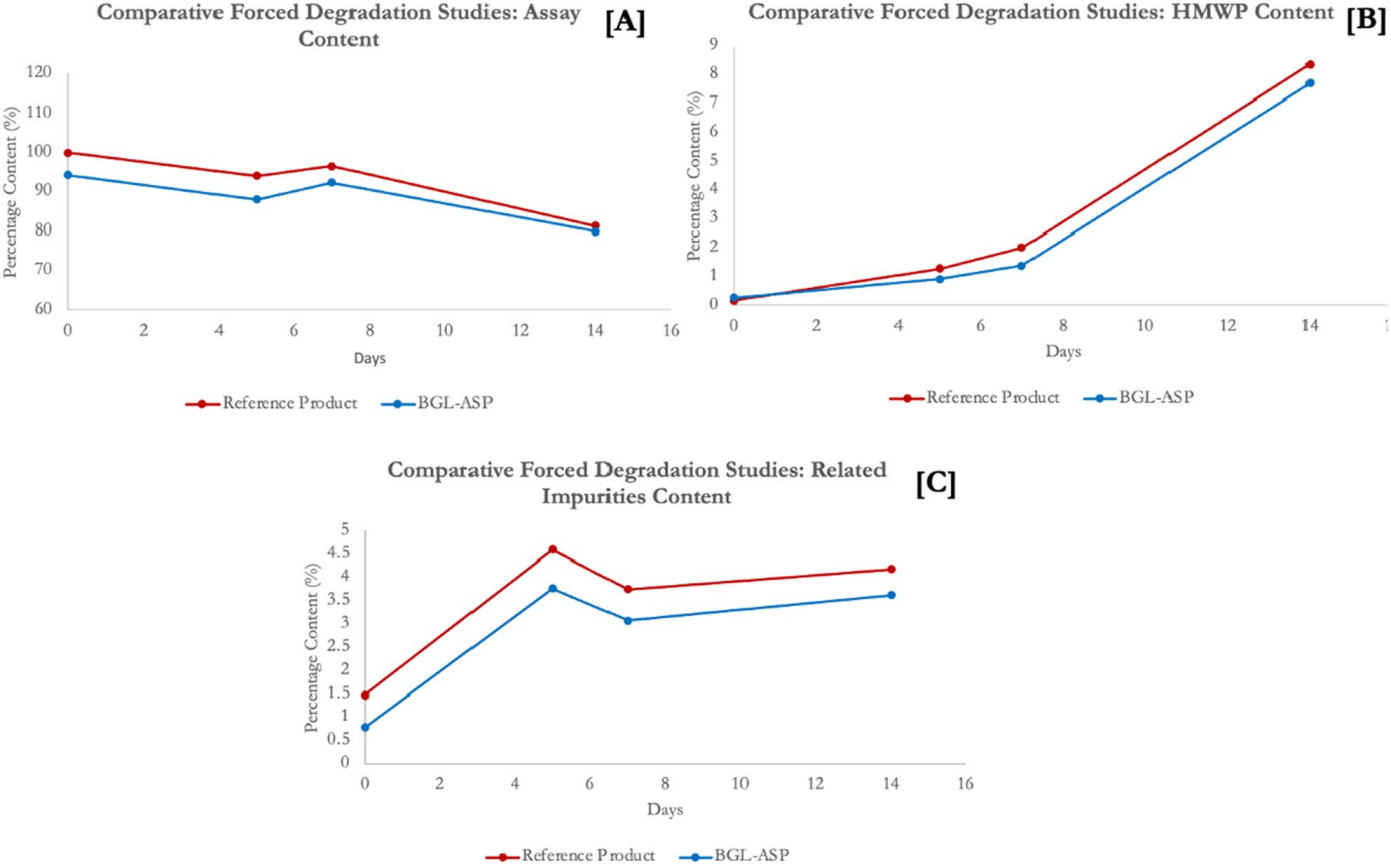
Forced degradation studies. Graphical Representation for in-use stability studies of BGL-ASP. The panel (**A**)–(**C**) demonstrate the assay, HMWP and related impurity content, respectively. The data is documented for a single batch of BGL-ASP and reference product.

The accelerated, in-use and forced degradation studies of BGL-ASP were performed in comparison with reference product. The assay, related impurities and HMWP content are attributes of insulin aspart that tend to change over a period. The graphs for these attributes are recorded in the Figs. 17, 18 and 19.

The rate change profile for assay, related impurities and HMWP content at accelerated, in-use and forced degradation conditions are found to be highly similar for BGL-ASP and reference product, thus confirming the equivalence between BGL-ASP and reference product.

#### Process related impurities of insulin aspart

The design of the manufacturing process ensures that the process related impurities are effectively removed at varied process stages. A significant reduction in process related impurities was observed at the capture chromatography stage. The process related impurities were mapped throughout the entire manufacturing process, thus providing a detailed understanding of the process capabilities. BGL-ASP was tested for a series of process related impurities tabulated in Table 12.

**Table 12:** Process related impurities of BGL-ASP.

Process related impurities of insulin aspart	Impact	Complies as per established safety limits
Bacterial endotoxin	Safety and immunogenicity	✓
Residual solvent content		✓
Residual single chain precursor		✓
Residual host cell protein		✓
Residual host cell DNA		✓
Residual enzyme content		✓
Residual moisture content		✓

The process related impurities were found to be within the established safety limits in BGL-ASP, thus confirming the product safety.

## Discussion

Physicians play a crucial role in prescribing biosimilar insulin to patients, and it is essential that the health care professionals are convinced about the quality, safety and efficacy of the drugs. Biologics such as insulins are less complex than monoclonal antibodies, however, the complexity is much higher than generic drugs which are typically smaller molecules with simpler structures. Thus, due to the complex biopharmaceutical manufacturing process which is unique for every protein molecule, the concerns of physician and healthcare would generally revolve across physicochemical biosimilarity, batch to batch consistency and similarity in impurity profiles. The onus to resolve these concerns lies with the manufacturer.

The manufacturing process of BGL-ASP is validated and is scientifically understood. The process parameters are evaluated and based on the impact on the critical quality attributes, the critical process parameters are identified. The design space for the critical process parameters is established and continuous monitoring of the process using in-process quality control checks ensures consistent manufacturing of BGL-ASP with established quality characteristics, thus maintaining batch to batch consistency. The similarity assessment studies were focused on identifying differences between the reference product and BGL-ASP and the data demonstrates high level of similarity for the critical quality attributes, thus providing the health care professional with an extensive data set related to the product’s purity, safety and potency.

A series of comprehensive structural, physicochemical and biological comparability (11) studies have been completed for BGL-ASP and reference product sourced from India, Switzerland, Denmark, Netherlands and USA. The data demonstrated high similarity for the structural and physicochemical quality attributes of BGL-ASP and reference product. The LC–MS data for the intact and reduced mass of insulin aspart and the peptide mass fingerprinting demonstrated that the primary structure was highly similar for BGL-ASP and reference product. A high level of similarity at secondary, tertiary and quaternary structural levels was established for BGL-ASP and reference product using state-of-the-art orthogonal spectroscopic tools such as NMR, FTIR, UV-CD, Intrinsic fluorescence and DSC. The physicochemical characterization focusses on impurity profiling, assay and excipient content determination and high similarity was observed for reference product and BGL-ASP. Stability studies such as real time, accelerated, in-use and forced degradation studies have been completed for BGL-ASP in comparison to reference product and the rate change profiles were found to be similar. The successful clinical study performed as per EMA/ICH guidelines generates adequate evidence to confirm that the product safety, efficacy and immunogenicity of BGL-ASP was highly similar to the reference insulin aspart product (CTRI/2019/04/018455).

The reference product is manufactured in genetically modified strain of *Saccharomyces cerevisiae* and BGL-ASP was manufactured in *E.coli*. The renaturation step was identified as a critical step in the manufacturing process of BGL-ASP and the structural characterization data confirmed a high similarity with reference product. The absence of glycosylated impurities in BGL-ASP proved advantageous with respect to purification strategy and the focus of the chromatographic separation was more towards the removal of deamidated and oxidized impurities.

## Conclusion

Based on the totality of evidence and the detailed physicochemical and structural characterization, it can be concluded that BGL-ASP is highly similar to reference product. BGL-ASP thus can provide a safe, efficacious, and cost-effective choice and access to clinicians and patients for controlling blood sugar levels.

This study provides a template for performing biosimilarity studies which involves identifying the QTPP by analysing multiple batches of reference product. The CQAs are identified based on the impact created on product quality, safety and efficacy. The biosimilarity limits and the criteria for establishing similarity are identified. The similarity assessment is completed through a series of comparative physicochemical, structural and biological characterisation studies.

## Data Availability

All data generated or analyzed during this study are included in this published article.
